# Ribonucleotide reductase M2B in the myofibers modulates stem cell fate in skeletal muscle

**DOI:** 10.1038/s41536-022-00231-w

**Published:** 2022-07-29

**Authors:** Wan-Jing Chen, I-Hsuan Lin, Chien-Wei Lee, Kiyoshi Yoshioka, Yusuke Ono, Yu-Ting Yan, Yun Yen, Yi-Fan Chen

**Affiliations:** 1 The Ph.D. Program for Translational Medicine, College of Medical Science and Technology, Taipei Medical University and Academia Sinica, Taipei, 11529 Taiwan; 2 TMU Research Center of Cancer Translational Medicine, Taipei Medical University, 11031 Taipei, Taiwan; 3 Center for Translational Genomics Research, China Medical University Hospital, Taichung, 404327 Taiwan; 4 Department of Muscle Development and Regeneration, Institute of Molecular Embryology and Genetics, Kumamoto University, Kumamoto, Japan; 5 Institute of Biomedical Sciences, Academia Sinica, Taipei, 11529 Taiwan; 6 Ph.D. Program for Cancer Molecular Biology and Drug Discovery, College of Medical Science and Technology, Taipei Medical University, 11031 Taipei, Taiwan; 7 Graduate Institute of Cancer Biology and Drug Discovery, College of Medical Science and Technology, Taipei Medical University, 11031 Taipei, Taiwan; 8 Cancer Center, Taipei Municipal WanFang Hospital, 116081 Taipei, Taiwan; 9 Center for Cancer Translational Research, Tzu Chi University, Hualien, Taiwan; 10 Graduate Institute of Translational Medicine, College of Medical Science and Technology, Taipei Medical University, 11031 Taipei, Taiwan; 11 International Ph.D. Program for Translational Science, College of Medical Science and Technology, Taipei Medical University, 11031 Taipei, Taiwan; 12 Master Program in Clinical Genomics and Proteomics, School of Pharmacy, Taipei Medical University, Taipei, 11031 Taiwan

**Keywords:** Gene expression, Muscle stem cells, Stem-cell niche, Regeneration

## Abstract

The balance among quiescence, differentiation, and self-renewal of skeletal muscle stem cells (MuSCs) is tightly regulated by their intrinsic and extrinsic properties from the niche. How the niche controls MuSC fate remains unclear. Ribonucleotide reductase M2B (Rrm2b) modulates MuSC quiescence/differentiation in muscle in response to injury. Rrm2b knockout in myofibers, but not in MuSCs, led to weakness of muscles, such as a loss of muscle mass and strength. After muscle injury, damaged myofibers were more efficiently repaired in the Rrm2b myofiber-specific knockout mice than the control mice, but these myofibers were thinner and showed weak functioning. Rrm2b-deleted myofibers released several myokines, which trigger MuSCs to differentiate but not re-enter the quiescent stage to replenish the stem cell pool. Overall, Rrm2b in the myofibers plays a critical role in modulating the MuSC fate by modifying the microenvironment, and it may lead to a possible strategy to treat muscle disorders.

## Introduction

Skeletal muscle constitutes nearly 40% of body mass and is primarily composed of multinucleated contractile muscle fibers. Muscle stem cells (MuSCs, also called satellite cells) lying between the sarcolemma and basal lamina of myofibers, are mitotically quiescent and express Pax7 and CD34^1,2^. MuSC activation and muscle regeneration are regulated by both intrinsic and extrinsic/microenvironmental factors that are affected by aging^3^. The intrinsic factors of MuSC activation include cell signaling, cell cycle modulators, key myogenic regulation factors (MRFs) and epigenetic signatures, while extrinsic factors are derived from myofibers, extracellular matrix, and other muscle resident cells and from systemic sources which trigger intrinsic alterations in MuSCs to govern local homeostasis^4^. Upon muscle injury and myofiber membrane disruption, MuSCs are activated, proliferate, differentiate, and fuse into myotubes. Proliferating MuSCs (committed myogenic progenitors) maintain the expression of Pax7 and induce the expression of MyoD and Myf5^5,6^. A subset of these proliferating MuSCs commits to differentiation through the expression of Myogenin and Myf6, accompanied by downregulation of Pax7 expression, ultimately producing myoblasts that exit the cell cycle and fuse to form regenerated myofibers with centrally located nuclei^7,8^. In contrast, another subset of proliferating MuSCs self-renews by inhibiting MyoD and reinducing quiescence to replenish the functional muscle stem cell pool to support future rounds of muscle repair.

Ribonucleotide reductase M2B (Rrm2B), also known as p53R2, is the small subunit of a heterotetrameric enzyme that catalyzes de novo synthesis of the deoxyribonucleotide triphosphates (dNTPs) required for DNA replication and repair; additionally, this process supplements the dNTPs produced by the mitochondrial dNTP salvage pathway^9–11^. Therefore, Rrm2b plays a critical role in producing dNTPs for DNA repair in the nucleus and mitochondria and DNA replication in mitochondria. We and others have demonstrated that Rrm2B is essential for mitochondrial DNA (mtDNA) synthesis, and defects in this gene cause mtDNA depletion syndromes and several organ disorders^12–15^. Mutations of the Rrm2B gene were shown to be responsible for severe muscle mtDNA depletion in patients with mitochondrial depletion syndrome^16^. Although mitochondria play a central role in skeletal muscle homeostasis, the role of Rrm2b in skeletal muscle remains unclear.

In this study, we determined the Rrm2b expression pattern in muscle regenerative process, revealed that Rrm2b may participate in the several stages of myogenesis and controls MuSC fate in muscle in response to injury. The specific knockout of Rrm2b in the muscle myofiber (a part of niche) but not in MuSCs compromised the regenerative capacity of MuSCs in mice. Lack of Rrm2b in the myofibers resulted in reduced mitochondria and an altered myokine profile that led to a decreased MuSC pool due to an impaired balance between differentiation and self-renewal of activated MuSCs, thus contributing to the loss of muscle mass and grip strength. Using a genetic approach, we identified a novel role of Rrm2b in muscle homeostasis, and animals with defective Rrm2b expression could serve as a disease model for investigating mitochondrial myopathy in mammals.

## Results

### The expression of Rrm2b in skeletal muscle during regeneration/repair

**Fig. 1:**
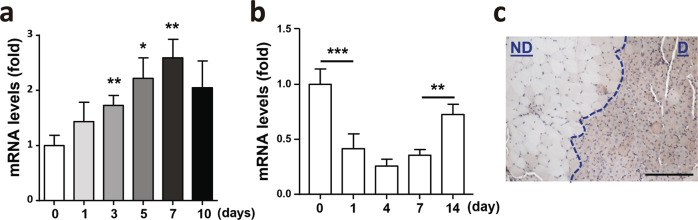
Rrm2b was upregulated at late stage of regeneration in wild-type skeletal muscle. **a** After induction of MuSC differentiation in vitro, the Rrm2b mRNA levels were significantly upregulated at days 3, 5, and 7. **b** Rrm2b expression levels in the skeletal muscle (gastrocnemius) of 5-month-old F/F mice at days 1, 4, 7, and 14 after damage. The values at day 0 served as the nondamaged controls, and 3–5 mice were in each group. **c** Rrm2b IHC expression in skeletal muscle (femoris) was higher in the damaged region 14 days after BaCl_2_ injection. ND, nondamaged region. D, damaged region showing centrally nucleated fibers. Scale bar, 100 µm. The mice were 3 months old. The results are presented as the mean ± SD. Statistical analysis of differences between the groups was performed by two-tailed, unpaired t-tests, and the *P*-values were calculated. Asterisks denote statistically significant changes from the control and are defined as *P < 0.05; **P < 0.01.

During the regenerative process, MuSCs proliferate at days 1–3 post-injury, show a peak of differentiation at days 3–5 post-injury, and show a peak of self-renewal at days 5–10 post-injury^17^. Our data revealed that isolated MuSCs had elevated Rrm2b expression after 3 days during in vitro differentiation (Fig. 1a). However, Rrm2b expression in wild-type mouse muscle was downregulated in the differentiation period and upregulated in the renewal period of regeneration (Fig. 1b). Increased Rrm2b expression was observed in damaged skeletal muscle compared to nondamaged skeletal muscle at 14 days after injury (Fig. 1c; Supplementary Fig. 1a). During muscle regeneration, the significant alteration of Rrm2b expression in myofibers was opposite to those in MuSCs. Whether Rrm2b plays a major role in muscle regeneration through MuSCs cell autonomous or non-cell autonomous need to be explored.

### Rrm2b deletion in myofibers, but not in MuSCs, reduces muscle mass and weakens muscle functions

**Fig. 2:**
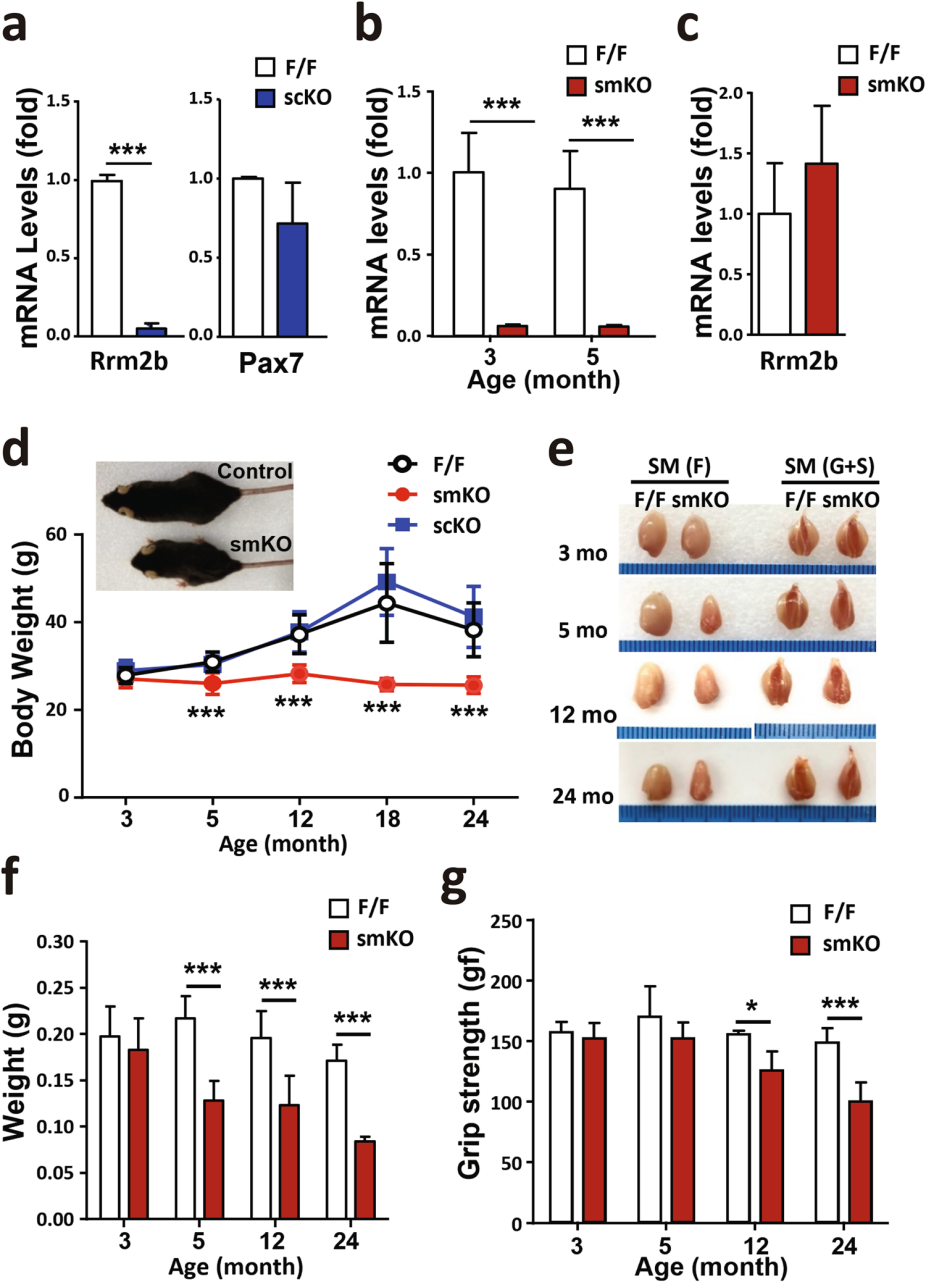
Rrm2b deletion in the myofibers leads to severe weakness in skeletal muscles. **a** Gene expression levels of Rrm2b and Pax7 were measured in the MuSCs of 3-month-old Rrm2b scKO mice; 3 mice were in each group. **b** Gene expression levels of Rrm2b were significantly downregulated in the skeletal muscle of the Rrm2b smKO mice; 5 mice were in each group. **c** Gene expression levels of Rrm2b were not reduced in the MuSCs isolated from the Rrm2b smKO mice; 3 mice were in each group. **d** Body weight observation of different mouse models from 3 to 24 months old; 10–15 mice were in each group. **e** Gross view of the skeletal muscles, femoris (F) and gastrocnemius and soleus (G+S) from the control (F/F) and Rrm2b smKO mice. **f** The muscle weight of the femoris in the Rrm2b smKO mice at all ages; 10–15 mice were in each group. **g** Grip strength of the Rrm2b smKO mice at 3, 5, 12, and 24 months of age; 6 mice were in each group. The results are presented as the mean ± SD. Statistical analysis of differences between the groups was performed by two-tailed, unpaired t-tests, and the *P*-values were calculated. Asterisks denote statistically significant changes from the control and are defined as *P < 0.05; **P < 0.01; ***P < 0.001.

To examine the role of Rrm2b in the regeneration/repair of skeletal muscles, we generated two knockout mouse models, MuSC-specific knockout (Rrm2b scKO) and myofiber-specific knockout (Rrm2b smKO) mice (Supplementary Fig. 1b). In the Rrm2b scKO mice, the expression levels of Rrm2b were significantly decreased by 95% in the MuSCs without affecting the expression of Pax7, suggesting that knockout of Rrm2b in MuSCs did not affect stem cell quiescence (Fig. 2a; Supplementary Fig. 1c); this model also did not show any loss of muscle mass or function (Supplementary Fig. 1d, e). In the Rrm2b smKO mice, the expression levels of Rrm2b were low in the myofibers but not in the MuSCs (Fig. 2b, c; Supplementary Fig. 1f). The expression of another small subunit of ribonucleotide reductase, Rrm2, was not changed in Rrm2b deleted model (Supplementary Fig. 1g). The loss of Rrm2b in myofibers caused growth retardation from 3 months old which resulted in a decrease in body weight and muscle weight with age (Fig. 2d–f; Supplementary Fig. 1h). Finally, the loss of muscle mass resulted in weakened muscle strength in the Rrm2b smKO mice at 12 and 24 months of age (Fig. 2g). Loss of Rrm2b in the myofibers may cause more severe myopathy than loss in the MuSCs.

### Increased centrally nucleated and thinner myofibers in the myofibers-specific Rrm2b knockout model

**Fig. 3:**
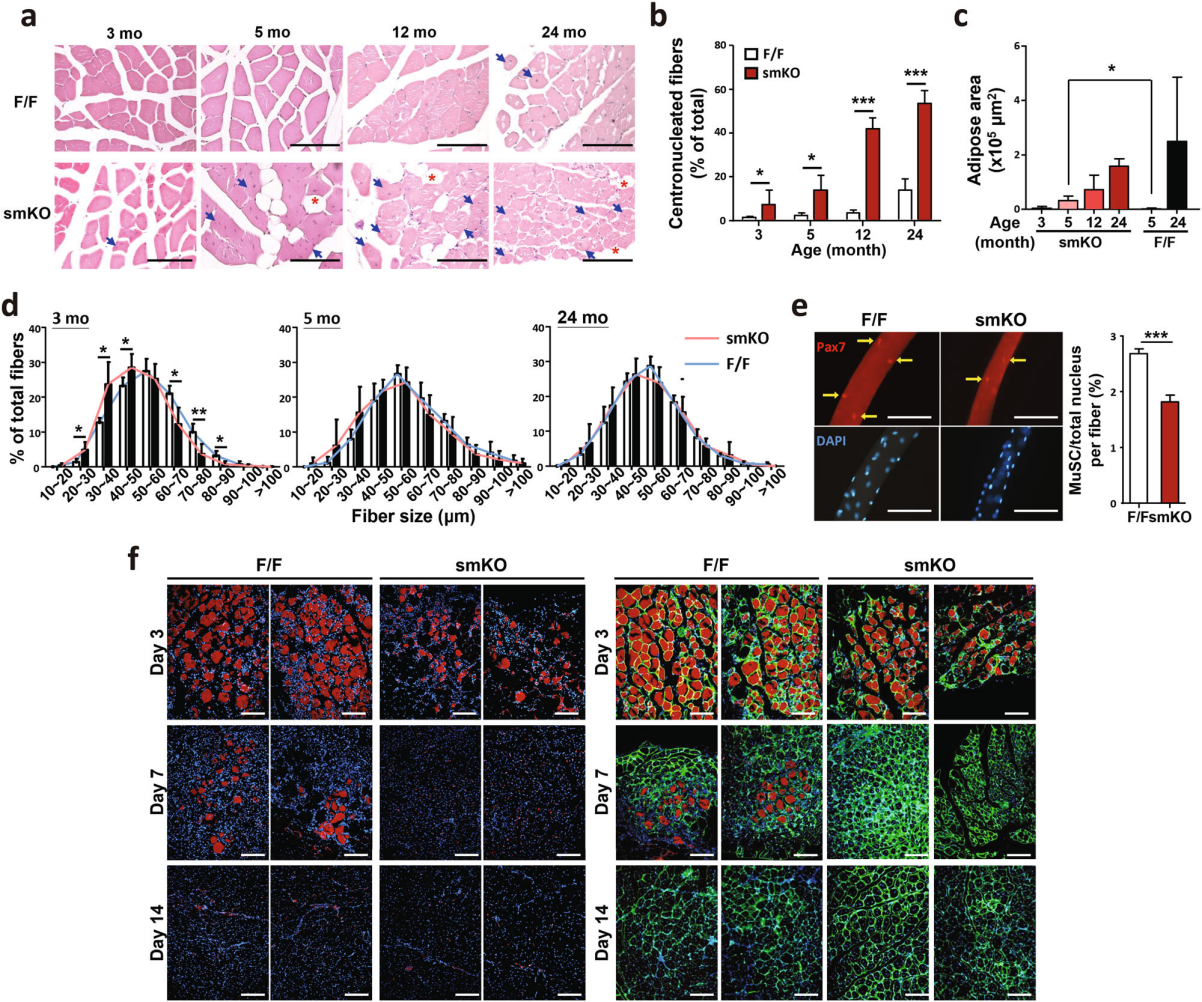
Promotion of the regeneration observed in the Rrm2b smKO mice. **a** H&E staining results of skeletal muscle from the Rrm2b smKO mice. Blue arrows indicate the centronucleated muscle fibers. Asterisk indicates adipose accumulation. Scale bar, 100 μm. **b** The percentage of centronucleated muscle fibers in the Rrm2b smKO mice. At least 6 photos (200×) from different fields were examined for each mouse. All single fibers were counted to determine whether the myonuclei were centrally located; 8 mice were in each group. **c** Quantification of the adipose area of the Rrm2b smKO mice. Five-month-old Rrm2b smKO mice showed a significant increase in the adipose area. The adipose area within the muscle fibers of the femoris was quantified with ImageJ software. The ratios were calculated by adipose area versus the whole femoris section, with 5 mice in each group. **d** Muscle fiber diameter of the femoris in the Rrm2b smKO mice. The diameter of 300 myofibers was measured in the femoris muscle of each mouse; 5 mice were in each group. **e** Immunostaining results of MuSCs (Pax7-positive) on isolated single myofibers in 3-month-old Rrm2b smKO mice; 15–20 myofibers in each mouse and 3 mice in each group were used. **f** EBD staining of TA muscle sections at 3, 7, and 14 days after injury. Red fluorescence indicates the damaged myofibers. Green fluorescence indicates immunostaining of laminin. Blue fluorescence indicates staining of DAPI. The mice were 5 months old. The results are presented as the mean ± SD. Statistical analysis of differences between the groups was performed by two-tailed, unpaired t-tests, and the *P*-values were calculated. Asterisks denote statistically significant changes from the control and are defined as *P < 0.05; **P < 0.01; ***P < 0.001. Scale bar, 100 μm.

Muscle regeneration is initiated shortly after damage, and centronucleated myofibers are formed and do not return to their original structure (nuclei positioned at their periphery). The number of centronucleated myofibers was elevated in skeletal muscle at old age. Surprisingly, loss of Rrm2b in the myofibers caused an age-dependent increase in centronucleated myofibers from 3 months of age (Fig. 3a, b). Adipose tissue, Perilipin (lipid droplet-associated protein)-positive, infiltration in skeletal muscle occurred earlier in the Rrm2b smKO mice than in the control (F/F) mice (Fig. 3c; Supplementary Fig. 2a). However, there was no damaged myofibers (EBD-positive) observed in the skeletal muscle of Rrm2b smKO mice which indicated spontaneous damage and cell death was not the primary issue to induce regeneration/repair in this mouse model (Supplementary Fig. 2b). The Rrm2b smKO mice had smaller myofibers than the F/F mice (Fig. 3d), but there was no significant difference in the population and distribution of fiber types (Supplementary Fig. 2c). We quantified the number of MuSCs in isolated single fibers and found that there were fewer MuSCs (Pax7 positive) in the Rrm2b smKO mice than the F/F mice (Fig. 3e); the number of MuSCs was obviously decreased in the Rrm2b smKO mice at old age (Supplementary Fig. 2d). However, the MuSC number in isolated single fibers from Rrm2b scKO mice was no significant changed compared to control group (Supplementary Fig. 2e). The number of MuSCs was elevated after muscle injury (Supplementary Fig. 2f, g) and the regenerated myofibers with intact boundary (Laminin- and eMyHC-positive and EBD-negative) almost replaced the damaged myofibers (EBD-positive) in the Rrm2b smKO mice at 7 days after injury (Fig. 3f; Supplementary Fig. 2h, i). Therefore, the efficiency of myofiber repair could be accelerated in Rrm2b smKO mice after muscle injury. The changes described above were not observed in the Rrm2b scKO mice (Supplementary Fig. 3a–c). Isolated MuSCs from Rrm2b smKO, scKO, and F/F could be well-differentiated and no difference among these mouse groups (Supplementary Fig. 3d). Loss of Rrm2b in the myofibers (a part of niche) decreased the MuSC pool and generated more centronucleated myofibers, which were thin and consistently accumulated in muscle over time, resulting in further weakening of muscle function; even so, after muscle injury, the muscle rapidly repaired damaged myofibers and generated more centrally nucleated myofibers in the Rrm2b smKO mice.

### Loss of Rrm2b in the myofibers weakens the regenerative capacity

**Fig. 4:**
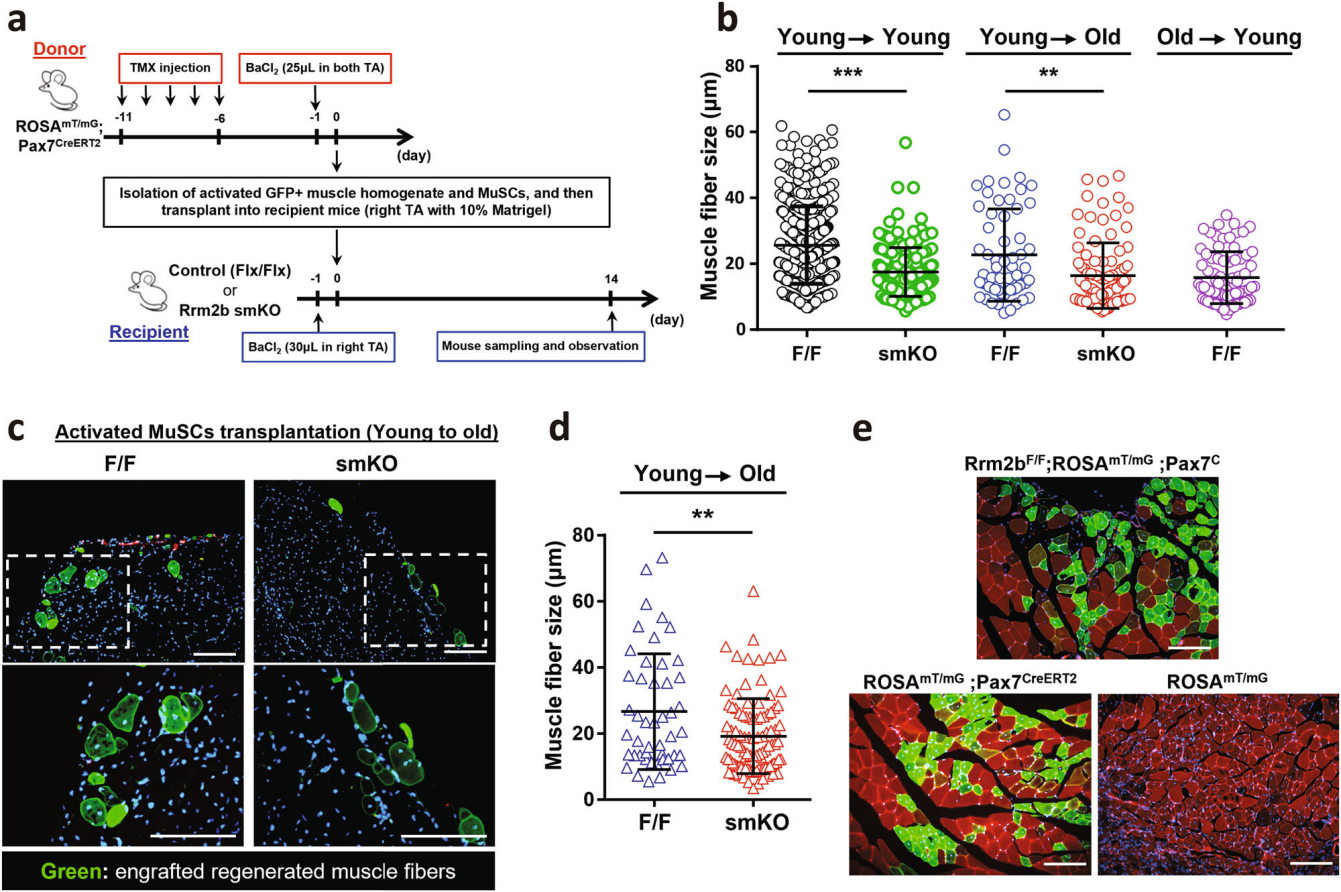
Myofiber-specific Rrm2b deletion weakens the regenerative capacity. **a** Donor and recipient mouse preparation timetable for muscle homogenate and MuSCs transplantation. **b** Quantification of engrafted myofiber size from homogenate transplantation between Rrm2b smKO and F/F mice. Each spot indicates one engrafted myofiber (myofibers with green fluorescence). **c** Representative images of engrafted myofibers in old recipient TA muscle. 14 days after MuSCs transplantation, activated MuSCs from young donors engrafted old recipient mice and differentiated into green myofibers with central nuclei. Scale bar, 100 μm. **d** Quantification of engrafted myofiber size from MuSCs transplantation between Rrm2b smKO and F/F mice. Each spot indicates one engrafted myofiber (myofibers with green fluorescence). **e** Histology of the gastrocnemius muscle from ROSA^mT/mG^, ROSA^mT/mG^;Pax7^CreERT2^, and Rrm2bF/F;ROSA^mT/mG^;Pax7^CreERT2^ mice at 14 days after damage. The mice were 5 months old, with 3 mice in each group. Scale bar, 100 μm. The results are presented as the mean ± SD. Statistical analysis of differences between the groups was performed by two-tailed, unpaired t-tests, and the *P*-values were calculated. Asterisks denote statistically significant changes from the control and are defined as **P < 0.01; ***P < 0.001.

To determine whether Rrm2b deletion in the myofibers indeed lead to the defects of muscle regeneration, we performed stem cell transplantation to examine the regenerative capacity of the Rrm2b smKO mice (Fig. 4a). The tamoxifen-inducible ROSA^mT/mG^; Pax7^CreERT2^ mouse model was used as the donor animal. After muscle injury, GFP-positive MuSCs were activated and fused to form regenerated myofibers, exhibiting bright green fluorescence in the tamoxifen-inducible ROSA^mT/mG^; Pax7^CreERT2^ mice (Supplementary Fig. 4a). Skeletal muscle homogenate, containing both MuSCs and soluble factors, can be used to repair damaged muscle tissues, which is more convenient, easier, and faster to prepare than isolated MuSCs^18^. We performed transplantation using muscle homogenate from young donors and again observed smaller regenerated myofibers in the Rrm2b smKO recipients than in the F/F recipients of the same ages (Fig. 4b; Supplementary Fig. 4b, c). When the muscle homogenate from an old donor (24 months) was transplanted into a young recipient, the regenerated myofibers in the recipients were also smaller. These data suggested that Rrm2b deletion in the myofibers led to regenerate smaller myofibers, and the factors within in muscle homogenate were not enough to rescue the Rrm2b defect in myofiber. Additionally, activated GFP-positive MuSCs (VCAM+/CD45−/CD31−/Sca1−) from young donor (3 months) mice were isolated and sorted according to the protocol shown in Supplementary Fig. 4d. Although the donor-derived GFP-positive MuSCs could differentiate and fuse into myofibers with green fluorescence in both F/F and Rrm2b smKO recipients, the regenerated GFP-positive myofibers were also smaller in the Rrm2b smKO recipients than the F/F recipients (Fig. 4c, d). More importantly, normal Rrm2b expression in the myofibers was sufficient for muscle regeneration and myofiber differentiation, even when Rrm2b was deleted in MuSCs (Fig. 4e). Our data demonstrated that loss of Rrm2b in the myofibers modified the stem cell microenvironment to impair the regeneration ability of MuSCs.

### Loss of Rrm2b in the myofibers promotes the differentiation of MuSCs

When MuSCs exit quiescence and re-enter the cell cycle, the expression levels of several genes involved in proliferation are upregulated. Efficient and lifelong regeneration of skeletal muscle requires a coordinated balance of MuSC potential for self-renewal, proliferation, and differentiation. Accumulating evidence indicates changes in cell polarity underlie asymmetric cell divisions whereby one daughter cell commits to differentiation while the other undergoes self-renewal and returns to quiescence. Maintenance of MuSCs pool relied on environmental factors to keep sufficient MuSCs in quiescent state. Furthermore, MuSCs adapt to environmental stress by either spontaneously activating to differentiation when the damage is not severe, or undergoing apoptosis and cell death when the stress is too severe^19^. Therefore, the ability of satellite cells to suitably balance quiescence, self-renewal, and commitment is crucial for muscle homeostasis.

**Fig. 5:**
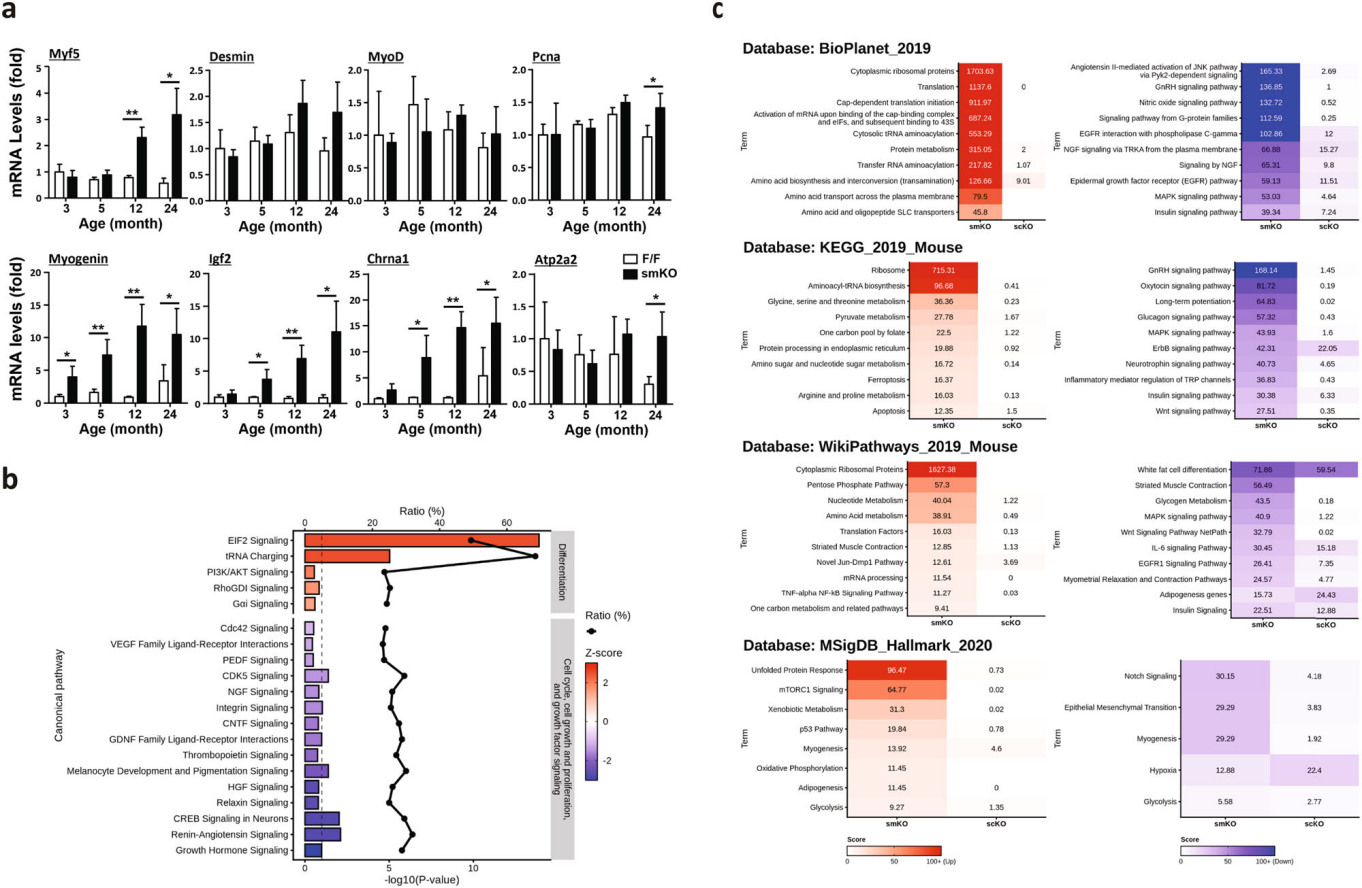
Rrm2b deletion drives MuSCs toward differentiation but not proliferation. **a** Quantitative data of mRNA expression of myogenic genes related to quiescence exit, proliferation, and differentiation in skeletal muscles (gastrocnemius) of the Rrm2b smKO mice at ages 3, 5, 12, and 24 months old using RT-qPCR analysis. Five mice were in each group. The data are presented as the mean ± SD. *P < 0.05; **P < 0.01. **b** The IPA analysis of RNA sequencing data. The red bar indicates the pathways related to cell differentiation. The blue bar indicates the pathways related to cell proliferation (cell cycle, cell growth and proliferation, and growth factor signaling). The selected pathways had a *P*-value < 0.05 and Z-score > 1 or <−1. **c** Enrichr analysis, including KEGG_2019_Mouse, WikiPathways_2019_Mouse, BioPlanet_2019, and MSigDB_Hallmark_2020. The top pathways were selected. The results were compared to those of the control group.

Our data suggested that loss of Rrm2b in the myofibers led to a decrease in MuSC number and an increase in the number of centronucleated myofibers, and the magnitude of these changes increased with age. Damaged myofibers were more quickly repaired in the Rrm2b smKO mice after muscle injury. Therefore, it is critical to understand when and how Rrm2b is involved in the regenerative process. The expression of genes related to myogenesis, including quiescence exit, proliferation, differentiation and return to quiescence, was changed in the Rrm2b smKO mice. Surprisingly, the genes related to myoblast differentiation, such as Myogenin, Igf2, and Chrna1, were significantly overexpressed from 3 months of age (Fig. 5a). Pathway analysis of the RNA sequencing data analyzed using Ingenuity Pathway Analysis (IPA) showed enhancement of pathways for differentiation and decreases in pathways for the cell cycle, cell growth, and proliferation, and growth factor signaling (Fig. 5b). Moreover, enrichment analysis using Enrichr and four gene-set libraries, KEGG_2019_Mouse, WikiPathways_2019_Mouse, BioPlanet_2019 and MSigDB_Hallmark_2020, revealed enhancement of pathways for differentiation and decreases in pathways for the cell cycle, cell growth and proliferation, and growth factor signaling (Fig. 5c). Rrm2b depletion could impact the self-renewal capacity, which deteriorated with age, resulting in a decrease in MuSC number in the Rrm2b smKO mice.

### Signals from the niche control the stemness of MuSCs

**Fig. 6:**
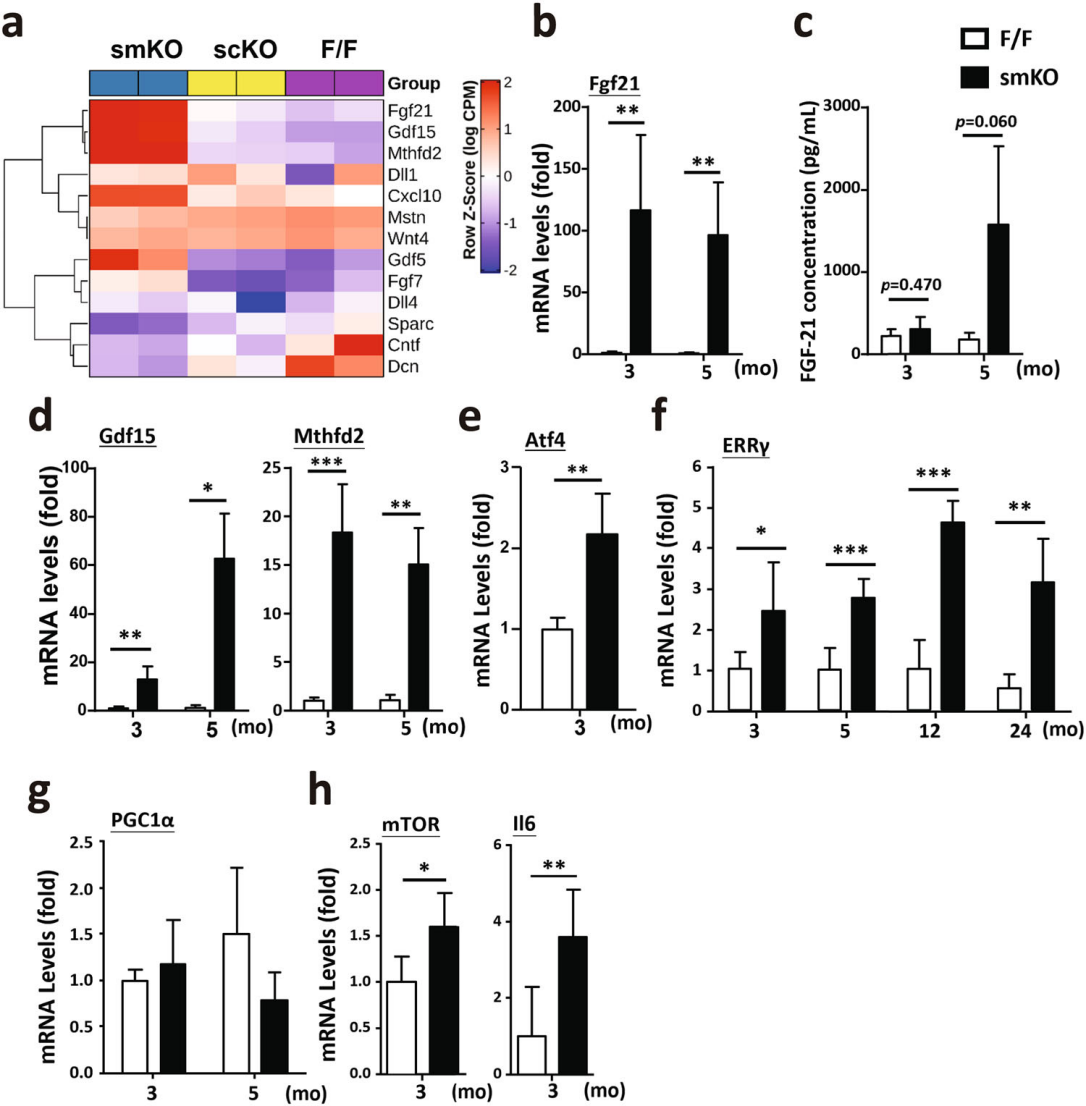
Myofibers modulate stem cell function by myokine secretion. **a** RNA sequencing data showed the expression of myokines in the gastrocnemius muscle of the Rrm2b smKO, scKO and F/F mice. **b** mRNA expression levels of Fgf21 were verified in muscle of the Rrm2b smKO mice. **c** Fgf21 levels in circulation were determined by ELISAs. **d** mRNA expression levels of Gdf15 and Mthfd2 were detected in muscle of the Rrm2b smKO mice. **e** mRNA expression levels of mTOR and Atf4 were detected in muscle of the Rrm2b smKO mice. **f** Gene expression levels of ERRγ were measured in muscle of the Rrm2b smKO mice at 3, 5, 12, and 24 months of age. **g** mRNA expression levels of PGC1α were detected in muscle of the Rrm2b smKO mice. In **b**–**g**, six mice were in each group. **h** mRNA expression levels of mTOR and Il6 were detected in MuSCs isolated from the Rrm2b smKO mice. In **h**, eight mice were in each group. The results are presented as the mean ± SD. Statistical analysis of differences between the groups was performed by two-tailed, unpaired t-tests, and the *P*-values were calculated. Asterisks denote statistically significant changes from the control and are defined as *P < 0.05; **P < 0.01; ***P < 0.001.

The stem cell niche in skeletal muscle tissue is responsible for the structural and functional maintenance of quiescence during tissue homeostasis and for mediating structural remodeling and regeneration. To further elucidate the regulation of MuSCs in the niche, we identified myokines whose expression was significantly changed in the Rrm2b smKO mice but not in the Rrm2b scKO mice compared to the F/F mice. In our RNA sequencing data, Fgf21, Gdf15, Mthfd2, Cxcl10, Fgf7, and Gdf5 levels were upregulated and Mstn, Cntf, Dcn, and Sparc levels were downregulated in the Rrm2b smKO mice, and Dll1, Dll3, and Wnt4 were not significantly altered in the knockout mice (Fig. 6a; Supplementary Table 3). These signals of extrinsic factors had been reported to stimulate MuSCs to differentiate and fuse with damaged myofibers or form new myofibers^20,21^. Among these myokines, Fgf21 had the greatest change in expression level (smKO vs. F/F, log2-fold change = 10.38); indeed, after validation, the expression levels of Fgf21 were elevated in the Rrm2b smKO mice (Fig. 6b, c). Gdf15, a critical myokine, was also upregulated in the Rrm2b smKO mice (Fig. 6d). Another key myokine, Mthfd2, which can induce cell differentiation via the Akt/mTOR signaling pathway, also showed significantly upregulated expression^22^ (Fig. 6d; Supplementary Table 3). The upstream regulators of Fgf21, such as Atf4 and ERRγ, also showed upregulated expression in the smKO skeletal muscle, possibly through the PGC1α-independent signaling pathway (Fig. 6e–g). A series of signals upregulated the expression of mTOR and Il6, which promoted MuSCs differentiation^23^ (Fig. 6h). Thus, Rrm2b deletion in the myofibers enhanced the secretion of specific myokines which may change the regulation of MuSC differentiation, quiescence, and self-renewal by modifying the stem cell microenvironment.

### The Rrm2b smKO mice might be a disease model for mitochondrial myopathy

**Fig. 7:**
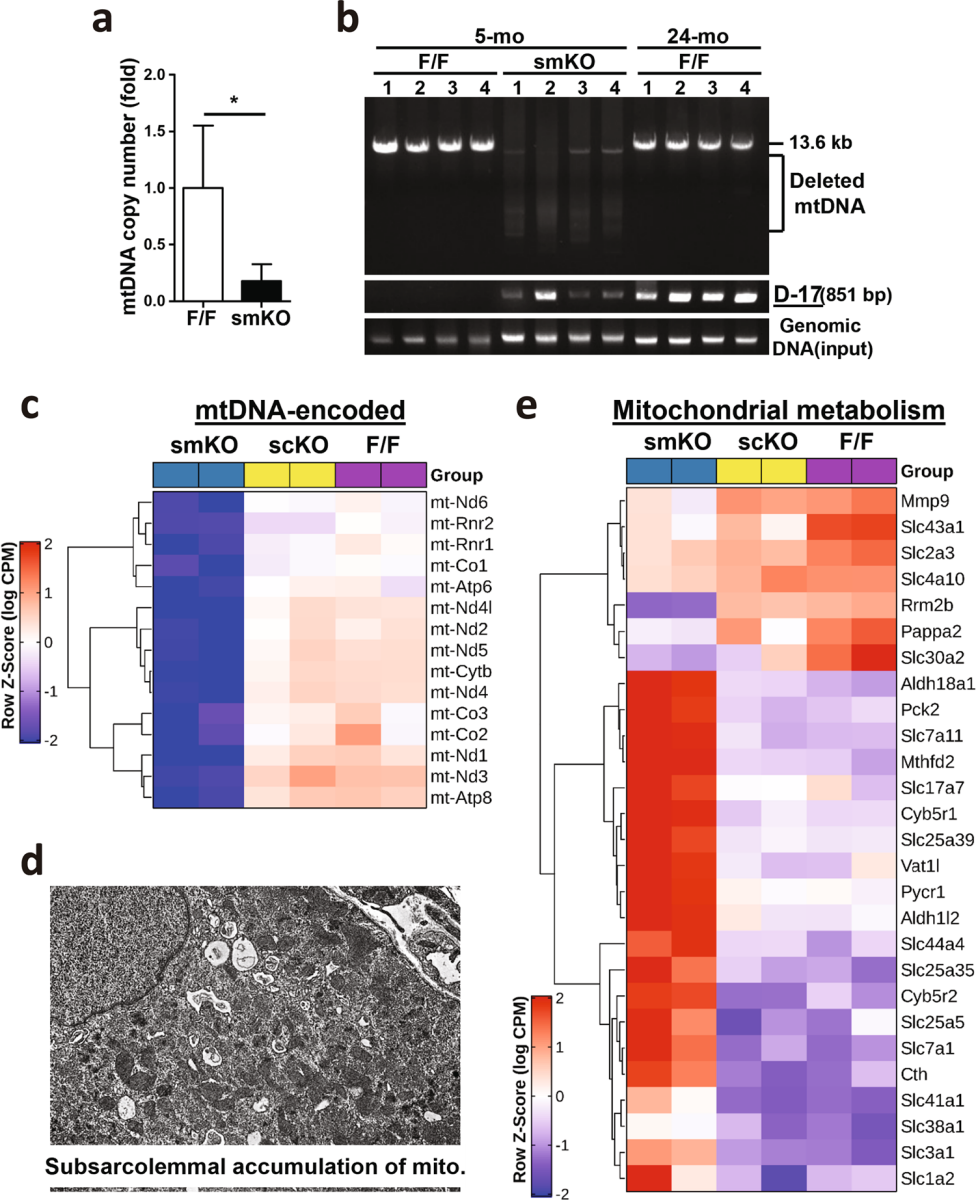
Decreases in mitochondria in skeletal muscles of the Rrm2b smKO mice. **a** mtDNA copy number was detected in the gastrocnemius muscle from 5-month-old Rrm2b smKO mice. Six mice were in each group. **b** Long-range PCR (13.6 kb) of mtDNA and detection of D-17 deletion (851 bp) using genomic DNA isolated from skeletal muscles. Genomic DNA was used for DNA input in PCR. **c** RNA sequencing data showed the expression of mitochondrial genomic DNA-encoded genes in the gastrocnemius myofibers of the Rrm2b smKO mice at 5 months of age. **d** Subsarcolemmal accumulation of mitochondria was observed in the type I muscle of the Rrm2b smKO mice at 5 months of age by transmission electron microscopy. **e** RNA sequencing data showed the expression of mitochondrial genomic DNA-encoded genes and mitochondrial metabolism-related genes in the type I myofibers of the Rrm2b smKO mice at 5 months of age. The results are presented as the mean ± SD. Statistical analysis of differences between the groups was performed by two-tailed, unpaired t-tests, and the *P*-values were calculated. Asterisks denote statistically significant changes from the control and are defined as *P < 0.05; **P < 0.01; ***P < 0.001.

Our previous reports indicated that Rrm2b is involved in maintaining the dNTP pool, inhibiting oxidative stress, and modulating mitochondrial metabolism^14^. Patients with myopathy were found to have genetic defects in RRM2B and decreased RRM2B expression^16^. We found that loss of Rrm2b in the myofibers upregulated the expression of Fgf21, which is a local and systemic messenger in mice and humans with mitochondrial myopathy through autocrine and paracrine mechanisms^24,25^. Rrm2b deletion in the myofibers significantly decreased the mtDNA copy number (Fig. 7a) and caused a high frequency of deletions in mtDNA (Fig. 7b). Our RNA sequencing data revealed that almost all mtDNA-encoded genes showed downregulated expression in the skeletal muscles of the Rrm2b smKO mice but not in the Rrm2b scKO mice (Fig. 7c; Supplementary Table 1). Subsarcolemmal accumulation of mitochondria, a hallmark of many mitochondrial disorders, was also observed in the Rrm2b smKO mice, further supporting the RNA sequencing results (Fig. 7d). The expression of mitochondrial metabolism-related genes showed major changes in the Rrm2b smKO mice compared to the Rrm2b scKO and F/F mice (Fig. 7e; Supplementary Table 2), but Rrm2b deletion appeared to cause little or no effect on the expression of genes involved in mitochondrial fusion-fission and biogenesis (Supplementary Fig. 5a, b). To investigate whether knockout of Rrm2b in myofibers affects mitochondrial functions in MuSCs, we measured the ex vivo mitochondrial respiration rate in the MuSCs isolated from the Rrm2b smKO mice and the F/F mice. Our assessments showed that the MuSCs isolated from the Rrm2b smKO mice had normal mitochondrial function (Supplementary Fig. 5c). Rrm2b deletion in the myofibers could induce a serial signaling pathway to increase the expression of Fgf21 or other myokines. Fgf21 could directly or indirectly trigger the pathogenesis of mitochondrial myopathy. Consequently, the Rrm2b smKO mouse model can be used to study the progression of mitochondrial myopathy.

## Discussion

**Fig. 8:**
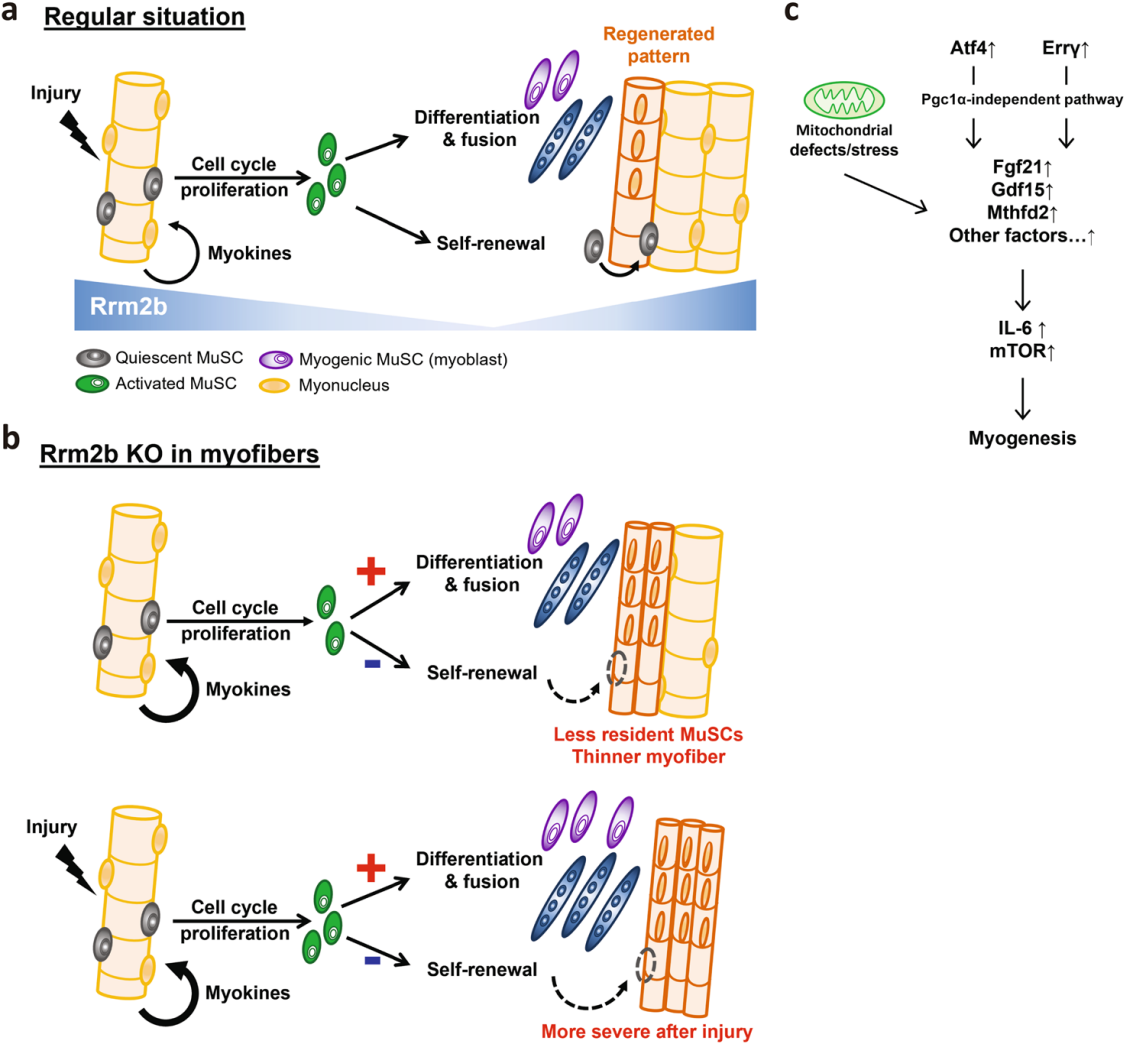
Rrm2b deletion in the myofibers promotes differentiation and arrests quiescence of stem cells when skeletal muscle is injured. **a** Under muscle damage, quiescent MuSCs enter the cell cycle, proliferate, and are then activated. Activated MuSCs either differentiate to form new myofibers or self-renew to re-enter the quiescent stage. **b** In the Rrm2b knockout myofibers, increased myokines continuously drive spontaneous MuSC activation and differentiation but inhibit self-renewal, resulting in a low number of MuSCs and myofiber weakness. **c** Our working hypothesis revealed that Atf4 and Errγ through PGC1α-independent signaling pathways and mitochondrial defects, upregulated expressions of Fgf21, Gdf15, Mthfd2 and other factors, which associated myogenesis in the Rrm2b-deleted myofibers.

Muscle injury, a kind of stress, drives quiescent MuSCs into the cell cycle for activation and proliferation. Cell cycle arrest can force activated MuSCs to differentiate and fuse to form new myotubes or fuse with damaged myofibers, thus regenerating the injured muscle. At the late stage of muscle regeneration, a subset of activated MuSCs re-enter the quiescent stage to refill the stem cell pool (Fig. 8a). In this study, we investigated whether Rrm2b has a critical role in skeletal muscle regeneration and the underlying mechanism. Notably, Rrm2b deletion in the myofibers disrupted the balance between MuSC differentiation and self-renewal after MuSC activation, and the loss of Rrm2b promoted differentiation and suppresses self-renewal, resulting in fewer MuSCs in muscles (Fig. 8b). Muscle injury could accelerate these phenotypes in the Rrm2b smKO mice (Fig. 8b). We hypothesize that Rrm2b plays a role in myofibers for regulating MuSC fate, specifically the impaired self-renewal of activated MuSCs resulting in pool exhaustion of stem cells. Additionally, our data revealed that Atf4 and ERRγ, through PGC1α-independent signaling pathways, upregulated Fgf21 and Mthfd2 expression in the Rrm2b-deleted myofibers, which altered MuSC stemness. Fgf21 and Mthfd2 are myokines, and when their expression levels are upregulated, they stimulate the differentiation of activated MuSCs with elevated expression of Il-6 and mTOR (Fig. 8c). Rrm2b deletion in the myofibers led to mitochondrial defects and dysfunctional muscle homeostasis, which caused continued damage in tissues even in the absence of injury caused by external forces. This phenomenon likely contributes to the progressive accumulation of centrally nucleated myofibers with age. We believe Rrm2b is part of the mechanism that modulates MuSC self-renewal and maintains quiescence in skeletal muscle. Without Rrm2b, the MuSC fate shifted toward myogenic differentiation instead of self-renewal, eventually exhausting the regenerative capacity of the muscle tissue.

Nearly half of adult mitochondrial diseases are caused by genetic defects in nuclear-encoded mitochondrial genes. Specifically, mutations in genes involved in the maintenance of mtDNA can lead to the accumulation of mtDNA deletions and/or a reduction in mtDNA copy number and further induce damage or injury due to mitochondrial dysfunction. Most proteins underlying mitochondrial depletion syndrome are involved in nucleotide metabolism^26^. Myofibrillar myopathy is characterized by variation in fiber size, rimmed vacuoles, internal nuclei, nuclear bags, basophilic and eosinophilic inclusions, fiber splitting, and necrotic and regenerating fibers. Kearns-Sayre syndrome, a rare disease belonging to progressive external ophthalmoplegia (PEO), is caused by heterozygous mutation in RRM2B. PEO patients typically show 5–20% cytochrome c oxidase (COX)-negative fibers in their muscles^27^. In familial PEO, mutations in the nuclear-encoded genes involved in respiratory chain function cause depletion of mtDNA and secondary deletions and point mutations of mtDNA^28^. In mitochondrial myopathies, FGF21 is induced and secreted from muscle for starvation-induced lipolysis, and this stress response is observed in both humans and mice^29^. We demonstrated that loss of Rrm2b in the myofibers caused severe myofibrillar myopathic features and mtDNA depletion and elevated expression of FGF21 in muscle and serum. These results indicated that Rrm2b has a critical function in maintaining muscle homeostasis, and loss of this gene can lead to muscle diseases.

We explored the importance of Rrm2b in skeletal muscle repair and regeneration and identified the Rrm2b tissue-specific knockout mouse model as a potential disease model for studying mitochondrial myopathy. Furthermore, Rrm2b may have a critical role in modulating MuSC fate and commitment. In summary, loss of Rrm2b in the myofibers led to increases in centronucleated myofibers by modifying the microenvironment around stem cells. A reduction in the muscle mass in the Rrm2b smKO mice further exacerbated the decrease in muscle strength with age. By analyzing the skeletal muscle transcriptome profiles, we showed that Rrm2b in the myofibers is part of the transcriptional network that can influence the expression of specific myokines, affecting muscle regeneration. In the development of prospective therapies for muscle disorders due to disease or aging, Rrm2b may be a potential therapeutic target for improving and/or rejuvenating muscle regenerative capacity.

## Methods

### Mouse models

B6.Cg-Tg(ACTA1-cre)79Jme/J (stock number: 006149) and B6.CgPax7^tm1(cre/ERT2)Gaka^/J mice (stock number: 017763) were purchased from The Jackson Laboratory. Rrm2b-floxed mice (Rrm2b F/F, Dr. Yun Yen Laboratory, City of Hope, US)^14^ were crossed with either Pax7^Cre-ERT2^ mice or HSA^Cre^ mice to generate Rrm2b myofiber-specific knockout (Rrm2b F/F; HSA-Cre, smKO) or satellite cell-specific knockout (Rrm2b F/F; Pax7-Cre^ERT2^, scKO) mice. Gt(ROSA)26Sor^tm4(ACTBtdTomato,EGFP)Luo^/J (ROSA^mT/mG^, stock number: 003474) mice were purchased from The Jackson Laboratory. The ROSA^mT/mG^;Pax7-Cre^ERT2^ mice were generated by crossing the ROSA^mT/mG^ mice with the Pax7^CreERT2^ mice. The Pax7-driven Cre^ERT2^ fusion gene was induced using tamoxifen (T5648, Sigma) in corn oil (C8267, Sigma) (at a concentration of 5 µL per g of 20 mg/mL) through daily intraperitoneal administration for 5 days following the instructions of The Jackson Laboratory. All animal protocols were approved by the Institutional Animal Care and Use Committee of Taipei Medical University and the National Defense Medical Center.

### Muscle injury

Mice were anesthetized by intraperitoneal injection of 2.5% avertin (T48402, Sigma)^30^ or by isoflurane. Mouse legs were shaved and cleaned with 70% alcohol. Muscle injury was induced by 25–50 µL of 1.2% BaCl_2_ in normal saline intramuscularly injected into the right tibialis anterior or gastrocnemius complex muscles. The contralateral (noninjected) leg served as a control.

### Genomic DNA extraction and genotyping by PCR

The mouse tail was digested in NTES buffer [100 mM NaCl, 50 mM Tris (pH 8.0), 50 mM EDTA, 1% SDS] with 200 µg/mL Proteinase K (1.24568.0500, Millipore) and then incubated at 55 °C overnight. Genomic DNA was extracted by phenol/chloroform and precipitated by 100% ethanol. The DNA pellet was resuspended in deionized water to a final concentration of 50–100 ng/µL for further genotyping. Genotyping was performed using Taq 2x Master Mix RED, 1.5 mM MgCl_2_ (5200300-1250, Ampliqon) or 2X RBC SensiZyme™ HotStart Taq Premix (RT008, RBCBioscience), and then, the PCR products were electrophoresed on 2 or 0.8% (w/v) agarose gels with Nancy-520 (01494, Sigma) and observed under ultraviolet light.

### RNA extraction and real-time quantitative PCR (RT-qPCR)

Muscle tissue was homogenized in TRIzol™ Reagent (15596018, Invitrogen™). RNA was extracted according to the manufacturer’s instructions and stored at −80 °C. cDNA was synthesized using a High-Capacity cDNA Reverse Transcription Kit (4368813, Applied Biosystems™). RT-qPCR was performed with specific primers (Supplementary Table 4) and TaqMan™ Universal Master Mix II, no UNG (4440040, Applied Biosystems™) on a QuantStudio3 Real-Time PCR system (Applied Biosystems) under standard conditions. All amplifications were carried out in triplicate for each RNA sample and primer set. The amount of total input cDNA was calculated using hypoxanthine-guanine phosphoribosyltransferase as an internal control.

### RNA sequencing data analysis

The raw sequencing reads were processed using Cutadapt [v 1.16^31^] to trim the adapter sequences and low-quality bases. The processed reads were then mapped to the mouse assembly GRCm38 using the STAR 2-pass mode [v 2.6.1a^32^]. Gene expression quantification was performed using RSEM [v 1.2.31^33^] with GENCODE annotations (release M18). Differential gene expression analysis was performed using the quasi-likelihood (QL) F-test method by edgeR^34^. The magnitude of gene expression changes is presented as log2-fold-changes, and a false discovery rate (FDR) of less than 0.05 was used as the threshold for statistical significance. Hierarchical clustering of selected groups of genes was performed in R, and the results were visualized as heatmaps using ComplexHeatmap^35^. Pathway enrichment analysis was performed using IPA (Qiagen, Inc., Valencia, CA, USA) and enrichR (https://CRAN.R-project.org/package=enrichR), which is an R Interface to the Enrichr resource^36^.

### mtDNA copy number

Genomic DNA was extracted from skeletal muscle of mice at 5 months old. qPCR was performed with a specific primer set and Power SYBR® Green PCR Master Mix (1905590, Applied Biosystems™) on a QuantStudio3 Real-Time PCR system (Applied Biosystems) under standard conditions. The mtDNA copy number was calculated by the quantification of nuclear DNA-encoded gene (18S rRNA) and mtDNA-encoded gene (Mitochondrially Encoded Cytochrome C Oxidase II, MTCO2) The primers for 18S rRNA detection were 5′-CTTAGAGGGACAAGTGGCGTTC-3′ and 5′-CGCTGAGCCAGTCAGTGTAG-3′, and the primers for MTCO2 detection were 5′-CCATAGGGCACCAATGATACTG-3′ and 5′-AGTCGGCCTGGGATGGCATC-3′. All amplifications were carried out in quadruplicate for each DNA sample and primer set.

### Protein extraction and Western blot

Tissue samples were homogenized in RIPA buffer (50 mM Tris at pH 7.4, 150 mM NaCl, 1% Triton X-100, 1% SDS, 1% deoxycholate) with complete protease inhibitor cocktail (04693132001, Roche) and denatured by boiling for 5 min. The extracted proteins were separated on SDS–polyacrylamide gel and electrotransferred to an Amersham™ Hybond® P Western blotting membrane, PVDF (10600023, GE Healthcare). The membranes were blocked with 5% (w/v) nonfat dry milk and 5% (w/v) bovine serum albumin (BSA, A7906, Sigma), incubated with primary antibody, washed, and then detected using a Visualizer Kit (WBKLS0500, Millipore). The antibodies used for detecting the target proteins are listed in Supplementary Table 5. Hsp70 was chosen as an internal control for each sample, and protein levels are expressed as the relative fold changes. All blots were derived from the same experiment and were processed in parallel. Un-cropped images of all blots in this study can be found in Supplementary Fig. 6.

### Single myofiber isolation

Extensor digitorum longus (EDL muscle) was isolated and digested with type I collagenase (LS004197, Worthington)^37^. After digestion, the single myofibers were carefully dissociated and the debris were cleaned. Isolated floating myofibers were cultured in BSA-coated plates to prevent attachment. Myofibers were cultured in DMEM consisting of 15% FBS, 1% chick embryo extract (C3999, USBiological) and 110 mg/L sodium pyruvate.

### Muscle stem cell (satellite cell) isolation and in vitro differentiation

To obtain satellite cells, Tibialis anterior (TA) muscle was digested in type II collagenase (LS004176, Worthington)^18^. After digestion, the muscle homogenate was dissociated with a 20 G needle, and filtered with 40 μm cell strainers. The muscle homogenate was resuspended with growth medium (GM) (DMEM supplemented with 20% fetal bovine serum, 0.5% chick embryo extract, 10 ng/mL basic fibroblast growth factor (Invitrogen), and 1% penicillin-streptomycin) and plated in collagen-coated dishes for incubating 12–16 h at 37 °C with 5% CO_2_ to remove most of debris. Then the supernatant which includes MuSCs was moved to a new collagen-coated dish for 3 h at 37 °C with 5% CO_2_ to remove remaining debris. Finally, the supernatant was seeded on a Matrigel (354230, Corning®)-coated dish, and isolated MuSCs can be observed on the next day after changing media. MuSCs isolated from muscles were cultured in GM at 37 °C with 5% CO_2_. To induce in vitro myogenic differentiation, isolated satellite cells (2 × 10^5^) were seeded in a well of 12-well plate. After cultured in GM for 24 h, the medium was switched to DMEM supplemented with 1.5% horse serum and 1% penicillin-streptomycin when we called it day 0 of differentiation. After another 24 h incubation, when it was day 1 of differentiation, the concentration of horse serum was added up to 15%, which is the DM (DMEM supplemented with 15% horse serum and 1% penicillin-streptomycin), and the satellite cells were cultured in DM for the rest of the days^38^.

### Muscle homogenate and MuSCs transplantation

For cost- and time-effectiveness, muscle homogenates were used as materials for transplantation since the isolation steps have been optimized as follow: Donor TA muscle was injured 1 day before collection. Donor TA muscle thinly chopped with scissors and digested using type II collagenase (LS004176, Worthington). Muscle homogenate was then dissociated from the myofibers with a 20 G needle, and the resulting cellular suspension was filtered with 40 μm cell strainers. The homogenate contains activated MuSCs, and each transplant was performed in a volume of 30 μL including 10% Matrigel (354230, Corning^®^)^18^. Recipient TA muscles of were also injured 1 day before they received transplants. Each TA muscle was transplanted with homogenate freshly isolated from one TA muscle of the donor mice. 14 days after transplantation, recipient TA muscles were collected for analysis. For isolation of the muscle stem cell (satellite cell) population, fluorescence-based cell sorting was performed according to the method described by Rando’s lab^39^. We obtained highly pure populations of activated muscle stem cells [VCAM (+) CD31(−) CD45(−) Sca1(−)] for subsequent transplantation experiments.

### Transmission electron microscopy

Mouse tissues were fixed in a mixture of glutaraldehyde and paraformaldehyde in phosphate buffer at pH 7.3. The samples were postfixed in 1% OsO4 and 1.5% potassium hexanoferrate and rinsed in cacodylate and 0.2 M sodium maleate buffers (pH 6.0). Dehydration and embedding were performed by the Core Facility Center of Taipei Medical University.

### Histopathological analysis

Skeletal muscle was collected and fixed within 10% neutral buffered formalin (Burnett) and subsequently dehydrated and embedded in paraffin. Tissue sections (4 μm) were subjected to hematoxylin-eosin (H&E) staining, Masson’s trichrome staining (Muto Pure Chemicals Co.), and immunostaining (IHC/IF) by standard procedures. IHC/IF staining was performed using paraffin-embedded skeletal muscle sections (3–4 μm). Sections were soaked in citrate antigen retrieval buffer (pH 6.1) (Dako, S1699) and heated in a microwave twice. The sections were then incubated with primary antibodies for 18–24 h at 4 °C, detected and visualized by a Dako REAL EnVision Detection System Kit (Dako, K500711). Single myofibers and satellite cells were fixed with 4% PFA. Immunostaining (IF) of satellite cells and isolated single fibers was performed with primary antibodies at 4 °C following blocking/permeabilization with phosphate-buffered saline (PBS) containing 0.3% Triton X-100 and 10% goat serum at 4 °C. Immunostained samples were visualized using appropriate species-specific Alexa® Fluor 488 or 568 fluorescence-conjugated secondary antibodies (Supplementary Table 5). Muscles were embedded in Tissue-Tek® O.C.T. compound (Sakura Finetek, 4583), frozen in liquid nitrogen-cooled 2-methylbutane (Sigma, M32631), stored at −80 °C, and cut into 7 µm thick cryosections with a cryostat (Sakura). Immunofluorescence analysis of muscle fiber type-specific MHC expression was performed with primary antibodies (Developmental Studies Hybridoma Bank, University of Iowa) against MHCI (BA-F8), MHCIIa (SC-71), MHCIIb (BF-F3) and MHCIIx (6H1) following a protocol previously described^40^. The primary and secondary antibodies used are listed in Supplementary Table 5. All slides were visualized with an IX73 microscope (OLIMPUS) using conventional widefield fluorescence microscopy as well as optical sectioning via structured-illumination fluorescence microscopy (Olympus). The microscope was equipped with red (excitation: BP 545/25 nm; emission BP 605/70 nm), green (excitation: BP 470/40 nm; emission BP 525/50 nm), and blue (excitation: BP 365/12 nm; emission LP 397 nm) filters, a QImaging Retina 3000 camera, and Q Capture Pro 7 software.

### Evans blue dye (EBD) injection

Evans Blue Dye (EBD), a non-permeating dye, is a reliable indication of sarcolemmal damage associated with muscle degenerative diseases since damaged myofibers with compromised sarcolemmal integrity allow the uptake of EBD^30,41,42^. EBD (Sigma, E2129) was dissolved in PBS at a concentration of 20 mg/mL, sterilized, and kept at 4 °C. Mice were intravenously injected with EBD solution (30 μL/10 g body weight) 3 h before muscle harvest. Blue color in the muscle indicated muscle damage. Red fluorescence in tissue sections indicated damaged myofibers.

### Detection of mtDNA deletion

Long mtDNA fragments (13.6 kb) and age-related deleted fragments (D-17) were generated using whole genomic DNA (or mtDNA alone) isolated from skeletal muscle as previously described^43^. The primers for the long PCR (13.6 kb) were 5′-GCCAGCCTGACCC ATAGCCATAATAT-3′ and 5′-ATTAATAAGGCCAGGAC CAAACCT-3′, and the primers for detection of D-17 deletion were 5′-GGAGATAAGTCGTAACAAGG-3′ and 5′-TGCTAGGAGAAGGAGAAATG-3′.

### Enzyme-linked immunosorbent assay (ELISA)

Mouse serum was harvested from both 3- and 5-month-old mice. There were at least 3 mice in each group. FGF-21 levels were measured by the Mouse FGF-21 Quantikine ELISA Kit (MF2100, R&D Systems) following the manufacturer’s instructions. The results were analyzed using a Synergy H4 Reader (BioTek Instruments).

### Statistical analysis

In all the figure panels, values are expressed as the mean with error bars that indicate standard deviations (SD) from at least three independent samples, if not stated otherwise. Statistical analysis of differences between the groups was performed by two-tailed, unpaired t-tests, and the *P*-values were calculated. Asterisks denote statistically significant changes from the control and are defined as *P < 0.05; **P < 0.01; and ***P < 0.001.

### Reporting summary

Further information on research design is available in the Nature Research Reporting Summary linked to this article.

## Supplementary information

Supplementary informaiton

REPORTING SUMMARY

## Data Availability

The RNA-seq data have been deposited in the ArrayExpress database at EMBL-EBI (http://www.ebi.ac.uk/arrayexpress) under accession number E-MTAB-11318. All data supporting this study are provided in this published paper and its Supplementary Information files. Any additional data are available from the corresponding author upon reasonable request.
