# Supplementary material for: Ribonucleotide reductase M2B in the myofibers modulates stem cell fate in skeletal muscle
Source: NPJ Regen Med. 2022 Jul 29;7:37. doi: 10.1038/s41536-022-00231-w (PMC9338274; doi:10.1038/s41536-022-00231-w)
Supplement: Supplementary file 1 — Supplementary informaiton [file 41536_2022_231_MOESM1_ESM.pdf]

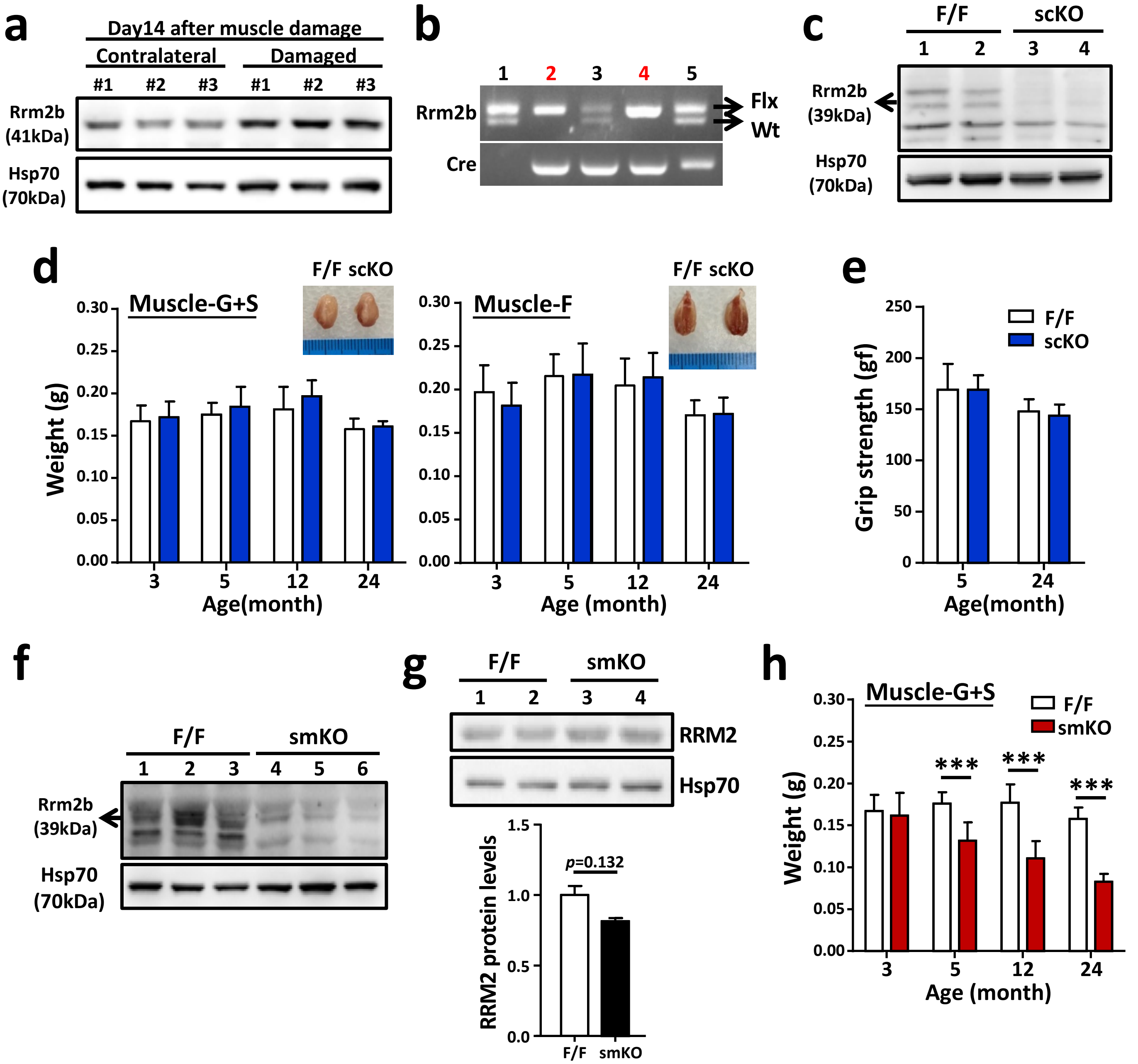

Supplementary Figure 1. Characterizations of the Rrm2b scKO and smKO mice. (a) The Rrm2b protein levels were significantly increased at 14 days after muscle damage in 3-month-old wild-type mice. **(b)** Genotyping result of tissue-specific knockout mouse models. Mouse no.2 and no.4, which showed Rrm2b floxed allele and Cre recombinase at the same time, were tissue-specific knockout mice. **(c)** Protein level of Rrm2b in MuSCs of 3-month-old Rrm2b scKO mice. **(d)** Muscle mass of gastrocnemius+soleus (G+S) and femoris (F) in Rrm2b scKO mice at different ages. 10-15 mice were in each group. **(e)** Grip strength of Rrm2b scKO mice at different ages. 6 mice were in each group. **(f)** The protein level of Rrm2b in the gastrocnemius muscle of 3-month-old Rrm2b smKO mice. **(g)** The protein level of Rrm2 in the gastrocnemius muscle of 3-month-old Rrm2b smKO mice. **(h)** Muscle mass of gastrocnemius and soleus in the Rrm2b smKO mice at different ages. 10-15 mice were in each group. F, femoris. G+S, gastrocnemius and soleus. The results are presented as the mean ± SD. **P*<0.05; ***P*<0.01; ****P*<0.001.

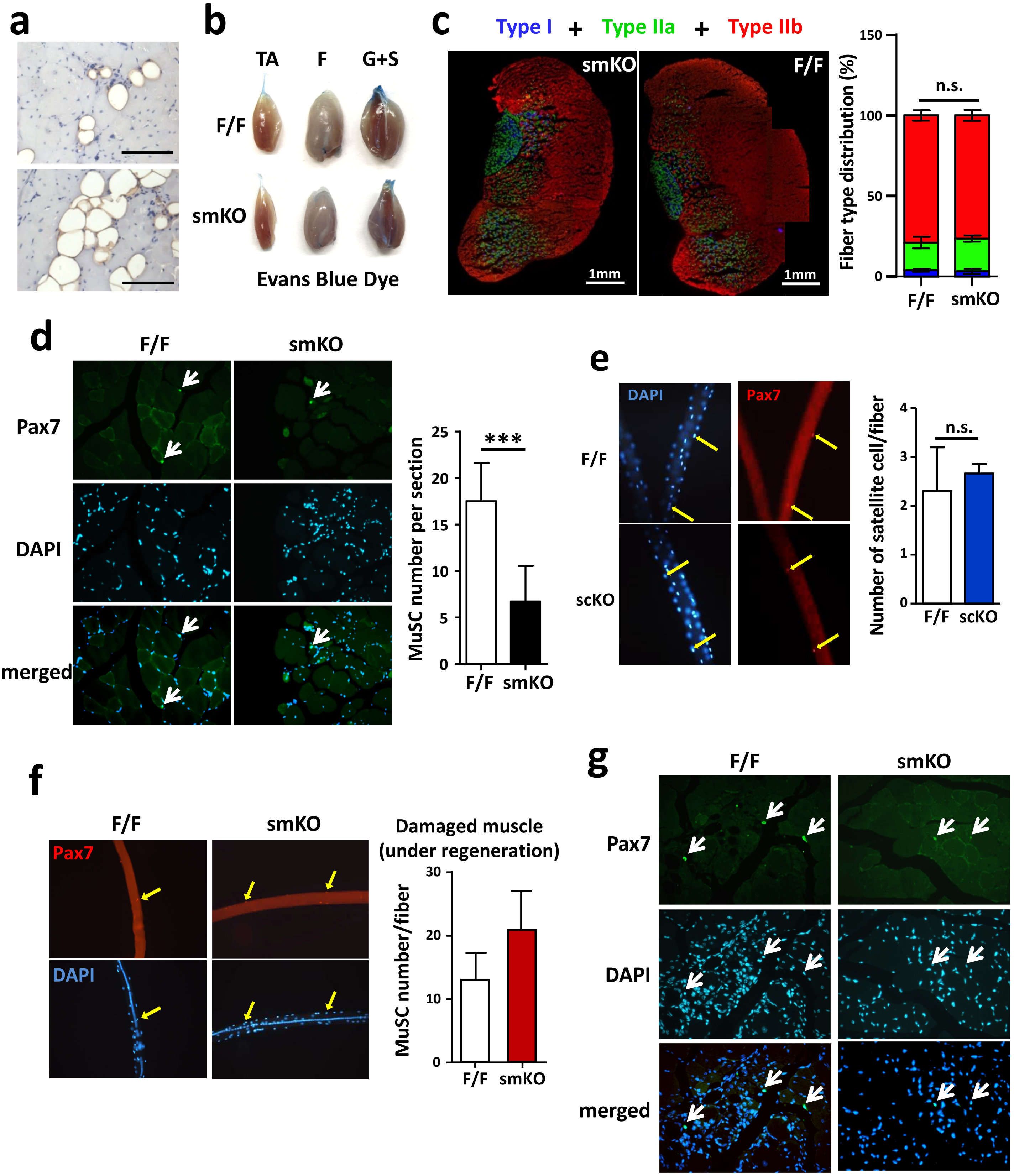
a
b
TA
F
G+S
F/F
smKO
Evans Blue Dye
c
Type I + Type IIa + Type IIb
smKO
F/F
1mm
1mm
Fiber type distribution (%)
150
100
50
0
n.s.
F/F
smKO
d
F/F
smKO
Pax7
DAPI
merged
MuSC number per section
25
20
15
10
5
0
***
F/F
smKO
e
DAPI
Pax7
F/F
scKO
Number of satellite cell/fiber
4
3
2
1
0
n.s.
F/F
scKO
f
F/F
smKO
Pax7
DAPI
Damaged muscle
(under regeneration)
MuSC number/fiber
30
20
10
0
F/F
smKO
g
F/F
smKO
Pax7
DAPI
merged

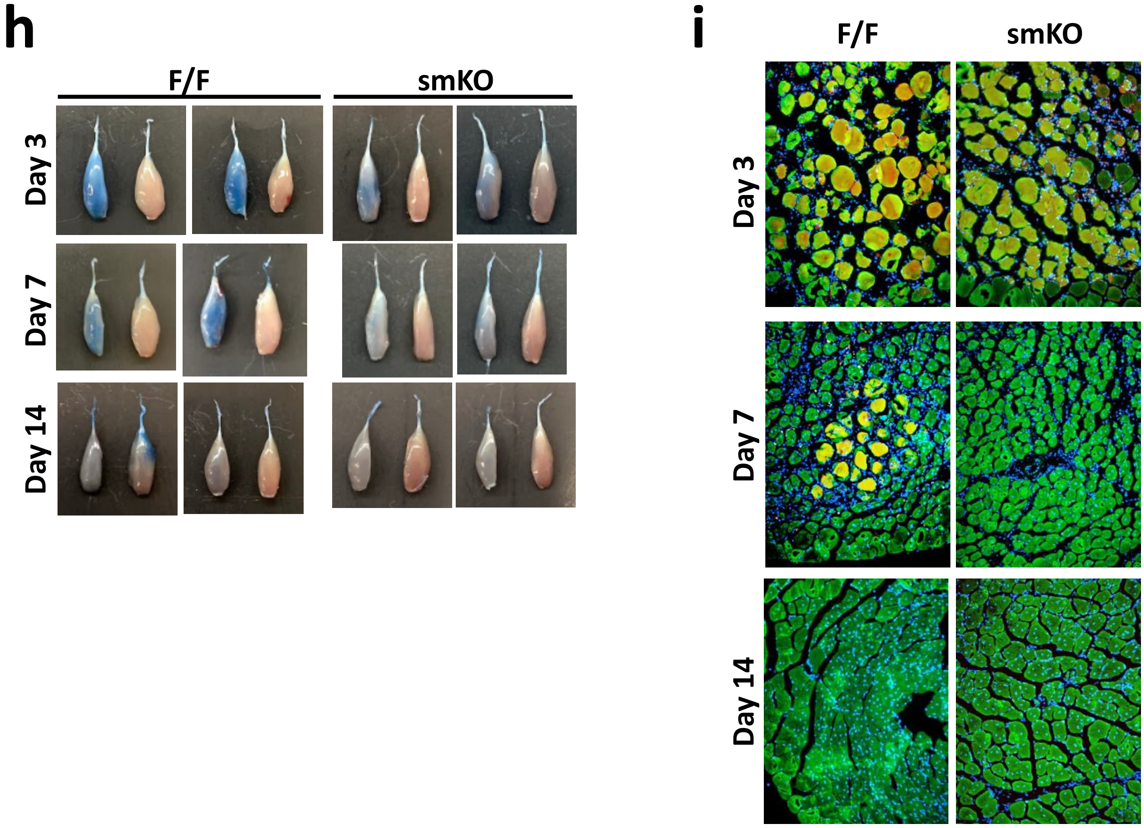

Supplementary Figure 2. The phenotypes of Rrm2b smKO and F/F mice. (a) The IHC staining for Perillipin, a protein coated on lipid droplet. **(b)** Skeletal muscles harvested from Evans Blue Dye-injected mice. Mouse age is 5 months old. **(c)** Immunostaining and quantification result of gastrocnemius complex muscle for distinguishing different fiber types in the Rrm2b smKO mice at 5 months old. Blue fluorescence indicates type I fiber using MHCI IHC staining. Green fluorescence indicates type IIa fiber using MHCIIa IHC staining. Red fluorescence indicates type IIb fiber using MHCIIb IHC staining. **(d)** MuSC staining in the skeletal muscle (femoris) of F/F and Rrm2b smKO mice at 24 months old. Arrows indicate MuSCs. Quantification of MuSC in femoris section of each mouse. 8 mice were in each group. **(e)** Qunatification of MuSCs on single myofibers isolated from Rrm2b scKO and F/F mice at 5 months old. Arrows indicate MuSCs. 3 mice were in each group. **(f)** Immunostaining results of MuSCs (Pax7-positive) on isolated single myofibers in 3-month-old Rrm2b smKO mice at 14 days after muscle injury; 15–20 myofibers in each mouse and 3 mice in each group were used. **(g)** MuSC staining in the skeletal muscle (gastrocnemius and soleus) of 5-month-old F/F and Rrm2b smKO mice 14 days after muscle injury. Arrows indicate MuSCs. 3 mice were in each group. **(h)** Isolated TA muscle at 3, 7 and 14 days after injury. Blue color indicates the damaged part in muscle. Mouse age is 3 months old. **(i)** EBD staining and immunostaining of embryonic heavy chain of Myosin (eMyHC) TA muscle sections at 3, 7 and 14 days after injury. Red fluorescence indicates the damaged myofibers. Green fluorescence indicates immunostaining of MyHC. Blue fluorescence indicates staining of DAPI. The mice were 5 months old.

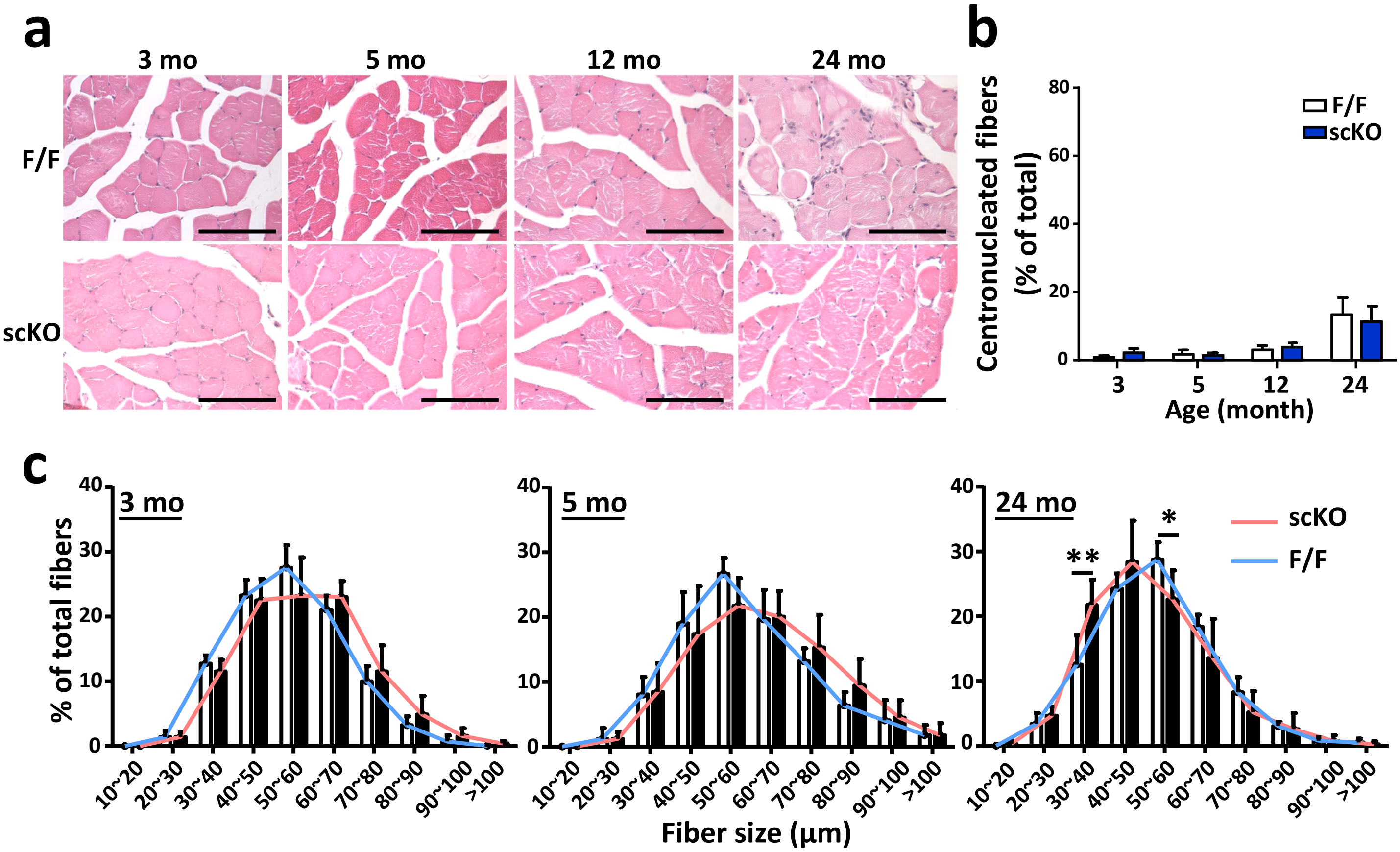
a
3 mo
5 mo
12 mo
24 mo
F/F
scKO
b
Centronucleated fibers (% of total)
F/F
scKO
Age (month)
c
3 mo
5 mo
24 mo
% of total fibers
Fiber size (μm)
scKO
F/F
10~20
20~30
30~40
40~50
50~60
60~70
70~80
80~90
90~100
>100

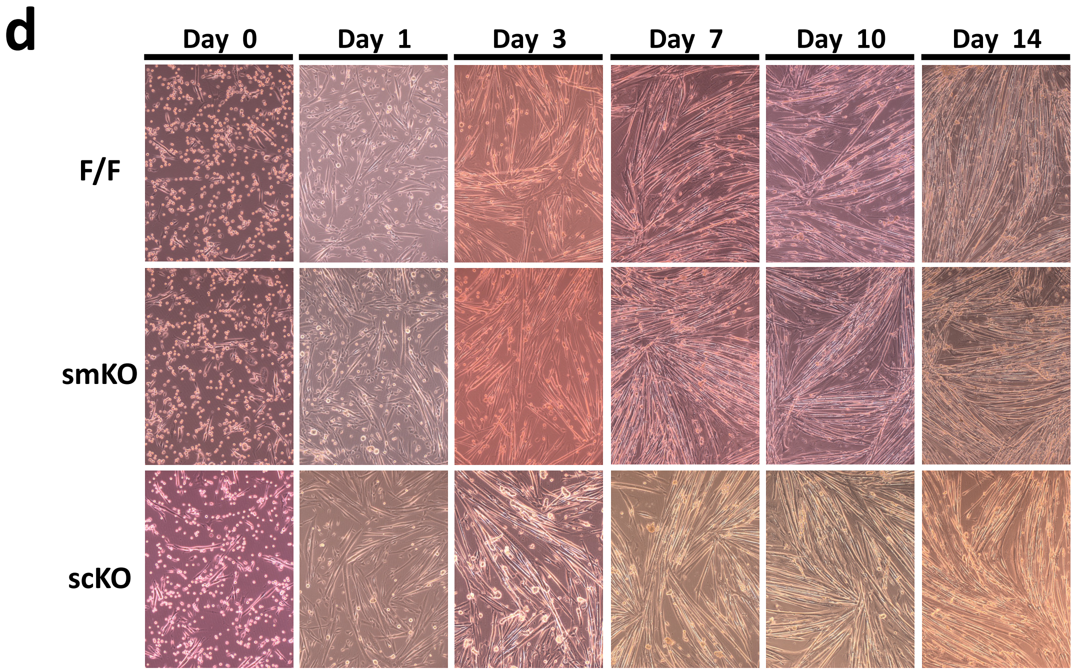

Supplementary Figure 3. The phenotypes of Rrm2b scKO and F/F mice. (a) H&E staining results of the skeletal muscle in Rrm2b scKO mice at 3, 5, 12, and 24 months old. Scale bar, 100μm. **(b)** The percentage of centronucleated muscle fibers in the Rrm2b scKO mice. 8 mice were in each group. **(c)** Muscle fiber diameter in the femoris of Rrm2b scKO mice at different ages. 5 mice were in each group. The results are presented as the mean ± SD. **P*<0.05 ; ***P*<0.01. **(d)** *In-vitro* differentiation of MuSCs from 5-month-old F/F, Rrm2b smKO, Rrm2b scKO mice. Isolated satellite cells ($2x10^5$) were seeded in a well of 12-well plate. On day 0, the media were switched from GM to DMEM supplemented with 1.5% horse serum and 1% penicillin-streptomycin. On day 1, the concentration of horse serum was added up to 15%, which is the DM (DMEM supplemented with 15% horse serum and 1% penicillin-streptomycin), and the satellite cells were cultured in DM for the rest of days.

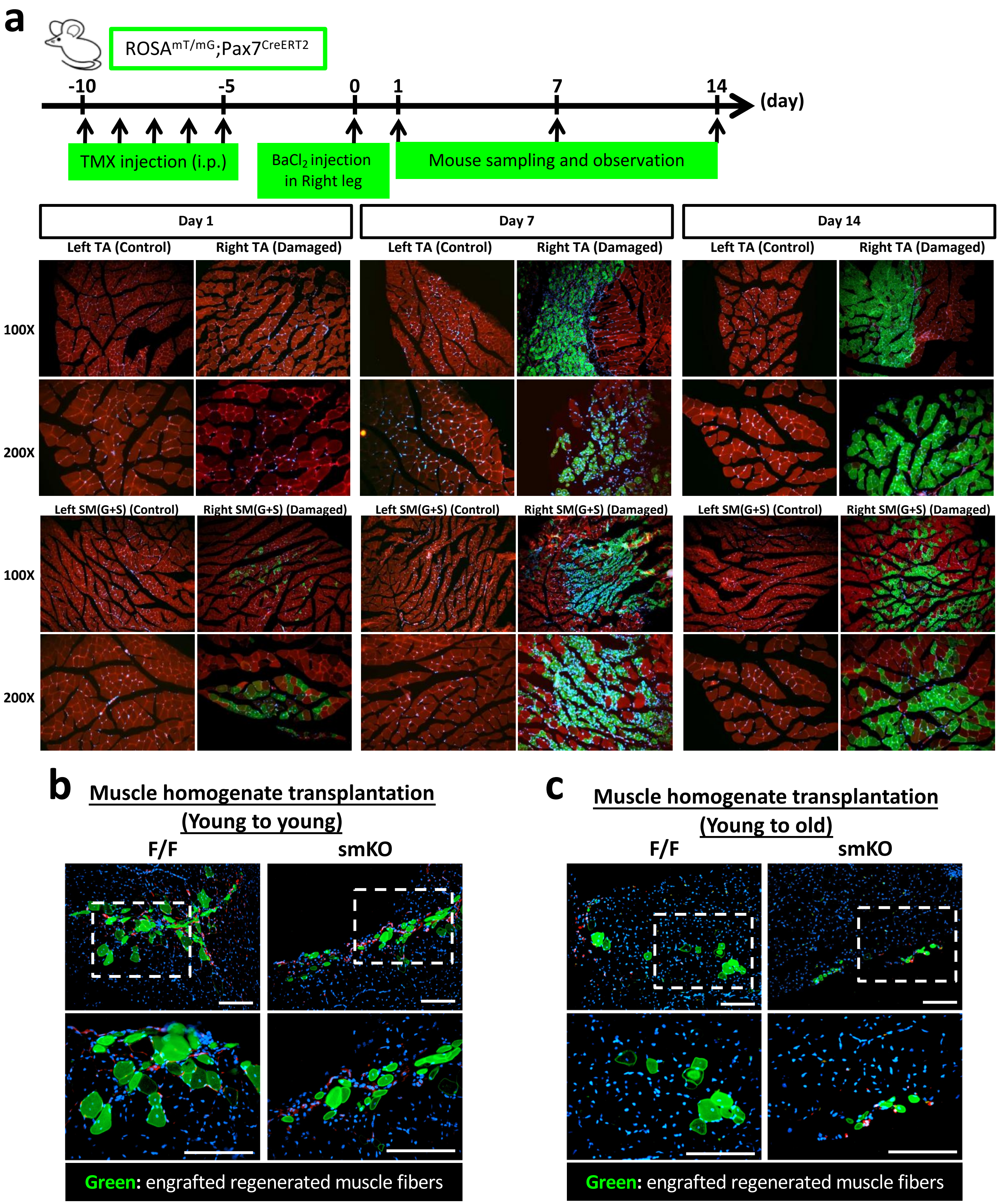

a
ROSA$^{mT/mG}$;Pax7^{CreERT2}
-10
-5
0
1
7
14
(day)
TMX injection (i.p.)
$BaCl_2$ injection in Right leg
Mouse sampling and observation
Day 1
Day 7
Day 14
Left TA (Control)
Right TA (Damaged)
100X
200X
Left SM(G+S) (Control)
Right SM(G+S) (Damaged)
b
Muscle homogenate transplantation (Young to young)
F/F
smKO
Green: engrafted regenerated muscle fibers
c
Muscle homogenate transplantation (Young to old)
F/F
smKO
Green: engrafted regenerated muscle fibers

d

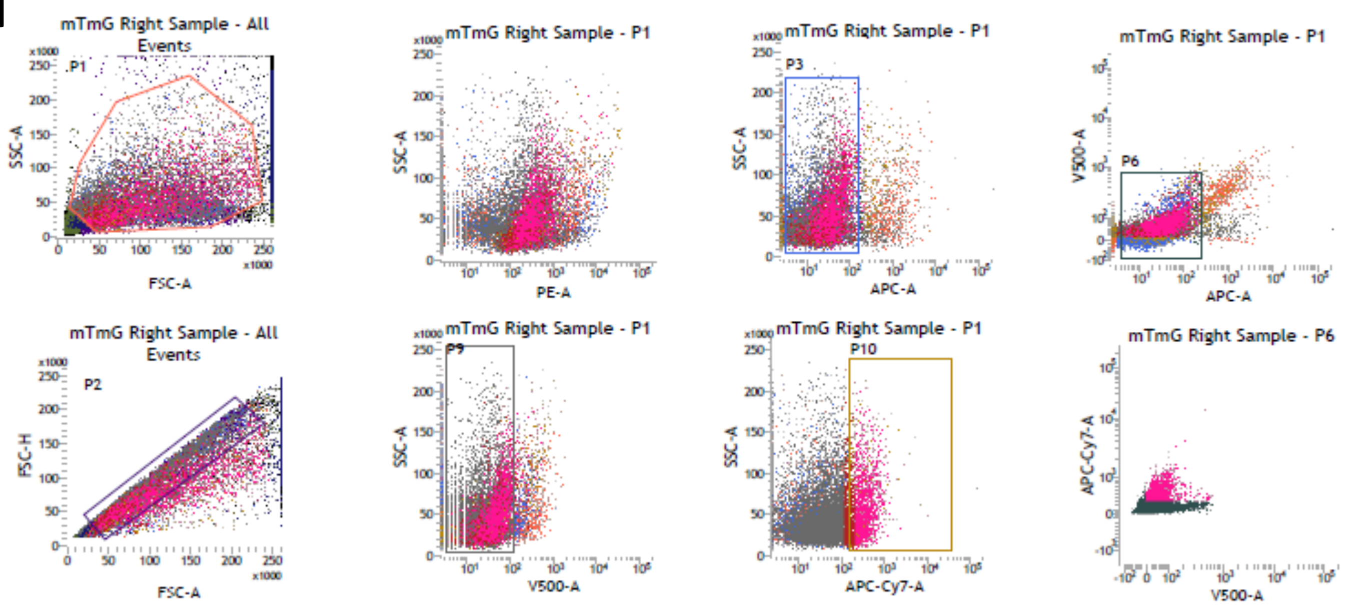

Statistics

Name	Events	% Parent	% Grandparent	% Total	FSC-A Mean	SSC-A Mean
CD106 Isptype:P8	218	1.77	1.36	0.60	120,942	96,349
mTmG Left Sample:P8	558	5.11	3.49	1.51	143,263	85,427
R-CD106 Isotype:P8	75	0.63	0.47	0.22	175,491	109,371
mTmG Right Sample:P8	1,676	15.06	10.48	5.29	134,006	59,018

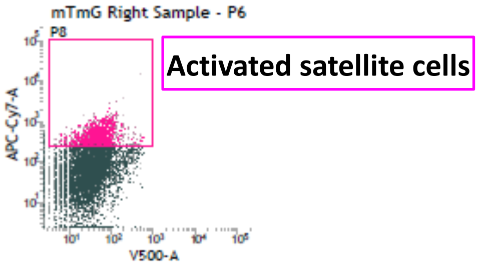

Supplementary Figure 4. Materials preparation for transplantation in skeletal muscles. (a) Establishment of ROSA$^{mT/mG}$;Pax7^{CreERT2} mice. After administration of tamoxifen to induce the expression of Pax7-Cre recombinase, the activated MuSCs turned to green fluorescence (EGFP) from red fluorescence (Tomato) and differentiated into regenerated fibers. Those regenerated fibers both in TA muscle and gastrocnemius complex muscles at 1,7, and 14 days after damage showed green fluorescent and centrally-located nuclei. **(b)(c)** Representative images of engrafted myofibers in young and old recipient TA muscle. Muscle homogenate was isolated from either old or young donor mice containing activated MuSCs with green-fluorescence. These activated MuSCs engrafted into recipient mice and differentiated to green myofiber with central nuclei at 14 days after homogenate transplantation. Scale bar, 100 µm. **(d)** Flow cytometry analysis of muscle homogenate from damaged TA muscle at 1 day after $BaCl_2$ injection. The percentage of activated MuSCs (VCAM$^+$/CD45$^-$/CD31$^-$/Sca1$^-$) was about 5%.

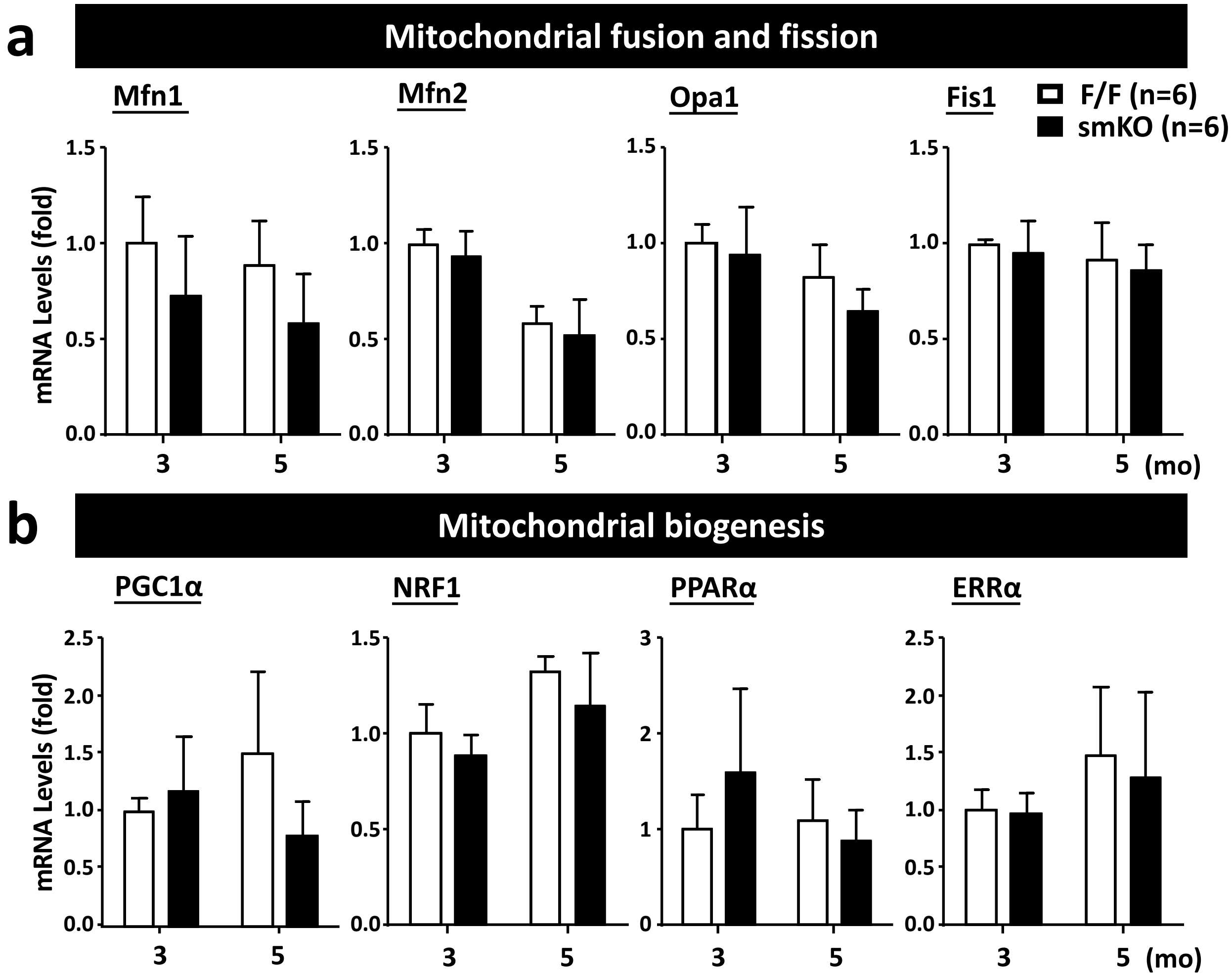
a
Mitochondrial fusion and fission
Mfn1
Mfn2
Opa1
Fis1
F/F (n=6)
smKO (n=6)
mRNA Levels (fold)
1.5
1.0
0.5
0.0
3
5
(mo)
b
Mitochondrial biogenesis
PGC1α
NRF1
PPARα
ERRα
mRNA Levels (fold)
2.5
2.0
1.5
1.0
0.5
0.0
3
2
1
0
3
5
(mo)

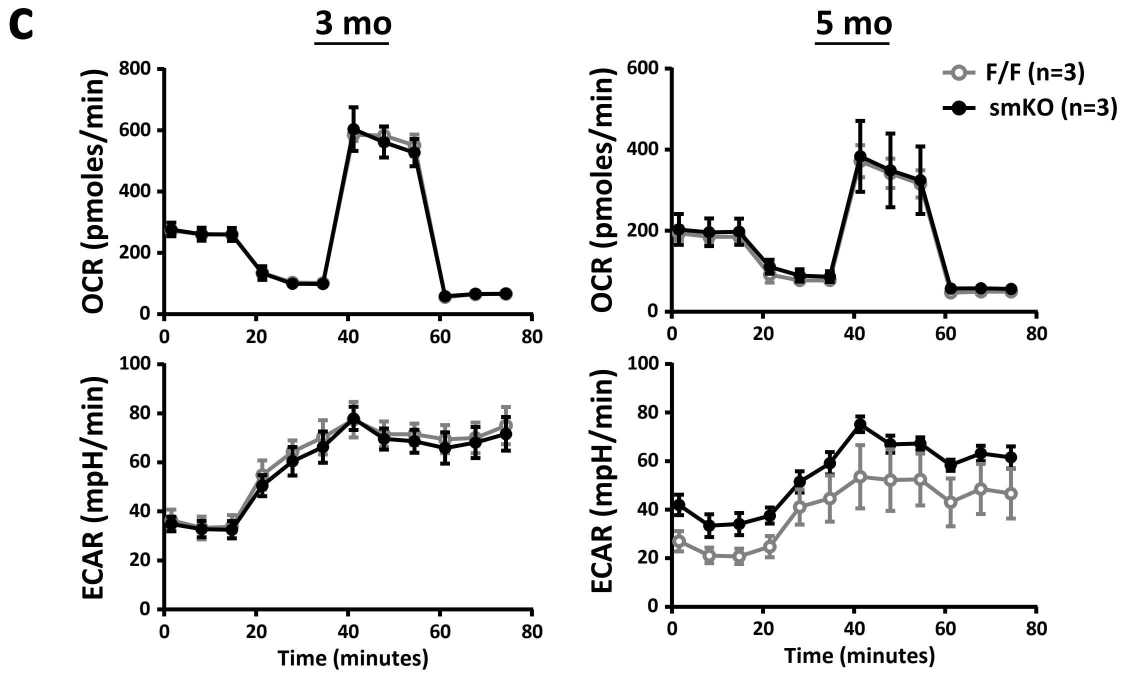

Supplementary Figure 5. Expression levels of genes for functional maintenance in the skeletal muscles. Quantitative data of mRNA expression of **(a)** the mitochondrial fusion and fission genes and **(b)** the mitochondrial biogenesis genes in the skeletal muscle of Rrm2b smKO mice at 3 and 5 months old using RT-qPCR analysis. **(c)** Mitochondrial functions of isolated MuSCs from Rrm2b smKO mice at 3 and 5 months old using Seahorse XFp analyzer. 3 mice were in each group. The results are presented as the mean ± SD.

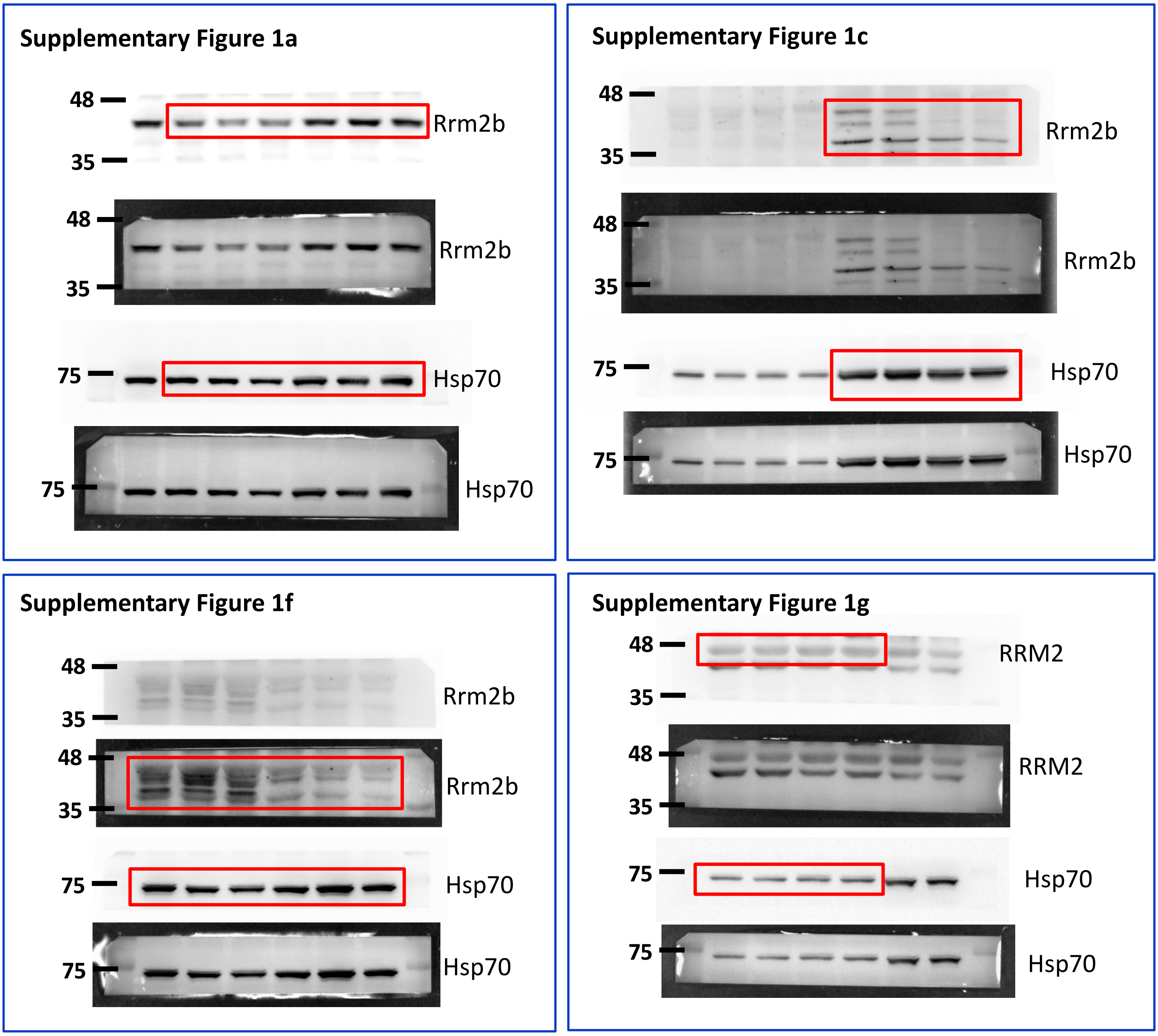

Supplementary Figure 6. Uncropped images of blots related to supplementary figures in this study. Red square frames indicate the representative bands used in the main text. All of the blots were captured twice to generate the merged images with molecular markers.

Supplementary Table 1

Supplementary Table 1. The gene expression levels of myokines in skeletal muscles of Rrm2b smKO mice.

chr	genes	name	logCPM	smKO_logFC	smKO_FDR
**chr7**	**ENSMUSG00000030827**	**Fgf21**	**1.84**	**10.38**	**8.38E-05**
**chr8**	**ENSMUSG00000038508**	**Gdf15**	**1.91**	**9.18**	**3.02E-06**
**chr2**	**ENSMUSG00000038259**	**Gdf5**	**-0.22**	**6.27**	**1.15E-04**
**chr6**	**ENSMUSG00000005667**	**Mthfd2**	**4.51**	**5.88**	**1.11E-05**
**chr5**	**ENSMUSG00000034855**	**Cxcl10**	**-1.33**	**2.4**	**1.41E-03**
**chr2**	**ENSMUSG00000027208**	**Fgf7**	**2.02**	**0.61**	**1.86E-02**
chr17	ENSMUSG00000014773	Dll1	1.83	0.11	8.36E-01
chr2	ENSMUSG00000027314	Dll4	2.7	0.02	9.79E-01
chr4	ENSMUSG00000036856	Wnt4	3.27	-0.26	2.45E-01
**chr11**	**ENSMUSG00000018593**	**Sparc**	**7.1**	**-0.54**	**1.54E-02**
**chr10**	**ENSMUSG00000019929**	**Dcn**	**7.88**	**-0.56**	**9.45E-03**
**chr19**	**ENSMUSG00000079415**	**Cntf**	**-0.04**	**-1.05**	**3.55E-02**
**chr1**	**ENSMUSG00000026100**	**Mstn**	**5.1**	**-1.13**	**1.02E-03**

Bold, smKO_FDR<0.05

Supplementary Table 2

Supplementary Table 2. The gene expression levels of genes that are encoded by mitochondrial genomic DNA in skeletal muscles of Rrm2b smKO mice.

Down-regulated expression of the mitochondrial genes					
chr	genes	name	logCPM	smKO_logFC	smKO_FDR
chrM	ENSMUSG00000064339	mt-Rnr2	17.43	-1.42	3.67E-05
chrM	ENSMUSG00000064337	mt-Rnr1	16.06	-1.66	3.41E-05
chrM	ENSMUSG00000064351	mt-Co1	14.31	-1.47	1.47E-05
chrM	ENSMUSG00000064370	mt-Cytb	12.69	-2.29	1.94E-06
chrM	ENSMUSG00000064363	mt-Nd4	12.59	-2.10	3.02E-06
chrM	ENSMUSG00000064341	mt-Nd1	12.44	-1.32	3.26E-05
chrM	ENSMUSG00000064367	mt-Nd5	12.33	-2.15	4.97E-06
chrM	ENSMUSG00000064345	mt-Nd2	12.03	-1.52	2.69E-05
chrM	ENSMUSG00000064357	mt-Atp6	11.06	-1.66	5.38E-05
chrM	ENSMUSG00000064358	mt-Co3	11.04	-1.97	2.27E-04
chrM	ENSMUSG00000064360	mt-Nd3	9.42	-1.88	9.06E-06
chrM	ENSMUSG00000064356	mt-Atp8	9.18	-2.05	3.02E-06
chrM	ENSMUSG00000064354	mt-Co2	8.96	-2.00	2.77E-03
chrM	ENSMUSG00000065947	mt-Nd4l	8.94	-2.33	7.57E-07
chrM	ENSMUSG00000064368	mt-Nd6	8.42	-1.92	7.27E-06

Supplementary Table 3

Supplementary Table 3. The gene expression levels of genes involved in mitochondrial metabolism in skeletal muscles of Rrm2b smKO mice.

Up-regulation of the gene expression involved in mitochondrial metabolism					
chr	genes	name	logCPM	smKO_logFC	smKO_FDR
chr3	ENSMUSG00000027737	Slc7a11	2.58	7.85	5.24E-05
chr6	ENSMUSG00000005667	Mthfd2	4.51	5.88	1.11E-05
chr7	ENSMUSG00000048065	Cyb5r2	-1.28	5.13	4.90E-05
chr17	ENSMUSG00000007034	Slc44a4	-3.94	3.91	2.67E-02
chr8	ENSMUSG00000046844	Vat1l	1.87	3.76	2.85E-03
chr19	ENSMUSG00000025007	Aldh18a1	3.10	3.66	4.64E-07
chr7	ENSMUSG00000070570	Slc17a7 (Vglut1)	-0.73	3.54	1.95E-03
chr17	ENSMUSG00000024131	Slc3a1	-2.34	3.38	6.33E-03
chr5	ENSMUSG00000041313	Slc7a1 (CAT-1)	3.58	3.25	4.63E-05
chr3	ENSMUSG00000028179	Cth	2.24	3.24	3.94E-04
chr11	ENSMUSG00000025140	Pycr1	2.38	3.21	9.43E-06
chr14	ENSMUSG00000040618	Pck2	3.30	3.12	2.83E-05
chr1	ENSMUSG00000026456	Cyb5r1	6.04	2.53	8.59E-07
chr10	ENSMUSG00000020256	Aldh1l2	2.82	2.41	1.53E-05
chr11	ENSMUSG00000018740	Slc25a35	2.15	1.62	5.43E-04
chr1	ENSMUSG00000013275	Slc41a1	6.13	1.55	1.93E-04
chr11	ENSMUSG00000018677	Slc25a39	4.88	1.50	7.80E-05
chr2	ENSMUSG00000005089	Slc1a2 (Eaat2)	-1.46	1.46	4.96E-02
chr15	ENSMUSG00000023169	Slc38a1	1.26	1.33	5.47E-03
chrX	ENSMUSG00000016319	Slc25a5	4.75	1.04	4.47E-03
**Down-regulation of the gene expression involved in mitochondrial metabolism**					
chr	genes	name	logCPM	smKO_logFC	smKO_FDR
chr6	ENSMUSG00000003153	Slc2a3	2.04	-1.09	9.24E-04
chr2	ENSMUSG00000027075	Slc43a1	3.06	-1.14	2.50E-03
chr2	ENSMUSG00000017737	Mmp9	0.61	-1.39	2.59E-02
chr1	ENSMUSG00000073530	Pappa2	-1.48	-1.62	3.49E-02
chr15	ENSMUSG00000022292	Rrm2b	3.91	-1.68	1.45E-06
chr2	ENSMUSG00000026904	Slc4a10	-0.13	-1.87	1.70E-02
chr4	ENSMUSG00000028836	Slc30a2 (ZnT2)	2.35	-2.25	2.95E-03

Supplementary Table 4

Supplementary Table 4. Primer list. Primers were designed and performed on analysis of mouse genotypes and quantification of specific gene expression.

Primers for genotyping using regular PCR

Official Symbol	Name	Sequence
Rrm2b	Ribonucleotide reductase regulatory TP53 inducible subunit M2B	F: 5'-ACTTACATCAGAGATCCAAAGAAAAG-3'
		R: 5'-TTGTTTCAATTGCATTAAATAAAAATTCC-3'
HSA-Cre	Human alpha-skeletal actin-Cre	F: 5'-CCGGTCGATGCAACGAGTGAT-3'
		R: 5'-ACCAGAGTCATCCTTAGCGCC-3'
mTmG	$ROSA^{mT/mG}$	F: 5'-CTCTGCTGCCTCCTGGCTTCT-3'
		R: 5'-TCAATGGGCGGGGGTCGTT-3'

Primers for qPCR with TaqMan™ system

Official Symbol	Name	Sequence
Hprt1	Hypoxanthine phosphoribosyltransferase 1	F: 5'-CCTCCTCAGACCGCTTTTT-3'
		R: 5'-AACCTGGTTCATCATCGCTAA-3'
Rrm2b	Ribonucleotide reductase regulatory TP53 inducible subunit M2B	F: 5'-GCAGCCAGTGATGGAATTG-3'
		R: 5'-GGAACCTGCACCTCCTGAC-3'
Hmox1	Heme oxygenase 1	F: 5'-AGGGTCAGGTGTCCAGAGAA-3'
		R: 5'-GTTCTGCTTGTTGCGCTCTA-3'
Nfe2l2	Nuclear factor, erythroid 2 like 2	F: 5'-CATGATGGACTTGGAGTTGC-3'
		R: 5'-CCTCCAAAGGATGTCAATCAA-3'
Atm	Ataxia telangiectasia mutated	F: 5'-TTCTCAAGCAGATGATCAAGAAGT-3'
		R: 5'-GACTTTGAGACCTGCATCATTCT-3'
APE1/Ref-1	Apurinic/apyrimidinic endonuclease 1	F: 5'-AAAGAAAGGTTTGGATTGGGTAA-3'
		R: 5'-CTGACCAGTACTGATGGGTGAG-3'
Foxo1	Forkhead box O1	F: 5'-CTTCAAGGATAAGGGCGACA-3'
		R: 5'-GACAGATTGTGGCGAATTGA-3'
Foxo3	Forkhead box O3	F: 5'-GCTAAGCAGGCCTCATCTCA-3'
		R: 5'-TTCCGTCAGTTTGAGGGTCT-3'
Foxo4	Forkhead box O4	F: 5'-AAGGACAAGGGTGACAGCAA-3'
		R: 5'-CTGTGCAAGGACAGGTTGTG-3'
Foxo6	Forkhead box O6	F: 5'-AAGGATAAAGGCGACAGCAA-3'
		R: 5'-GTGTGCAGCGACAGGTTG-3'
Keap1	Kelch like ECH associated protein 1	F: 5'-CTGCACTGAACTGCACCAG-3'
		R: 5'-GGCAGTGTGACAGGTTGAAG-3'

SOD1	Superoxide dismutase 1	F: 5'-CAGGACCTCATTTTAATCCTCAC-3'
		R: 5'-TGCCCAGGTCTCCAACAT-3'
SOD2	Superoxide dismutase 2	F: 5'-TCAATGGTGGGGGACATATT-3'
		R: 5'-AGCCTCCAGCAACTCTCCTT-3'
Catalase	Catalase	F: 5'-CCTTCAAGTTGGTTAATGCAGA-3'
		R: 5'-CAAGTTTTTGATGCCCTGGT-3'
Pcna	Proliferating cell nuclear antigen	F: 5'-CTAGCCATGGGCGTGAAC-3'
		R: 5'-GAATACTAGTGCTAAGGTGTCTGCATT-3'
Cdh1	Cadherin 1	F: 5'-AGTGTTTGCTCGGCGTCT-3'
		R: 5'-GCAAAGCCATGAGGAGACC-3'
Mfn1	Mitofusin 1	F: 5'-GCTGTCAGAGCCCATCTTTC-3'
		R: 5'-CTCCTGGGCTGCATTATCC-3'
Mfn2	Mitofusin 2	F: 5'-GGAGACCTCGAGTCCTTTTCA-3'
		R: 5'-ACAACTGGAACAGAGGAGAAGTTT-3'
Opa1	OPA1 mitochondrial dynamin like GTPase	F: 5'-CAGCTGGCAGAAGATCTCAA-3'
		R: 5'-TTATGAGCAGGATTTTGACACAG-3'
Fis1	Fission, mitochondrial 1	F: 5'-AGCTGGTGTCTGTGGAGGAT-3'
		R: 5'-ATTGCGTGCTCTTGGACAC-3'
Ppargc1α	Peroxisome proliferative activated receptor, gamma, coactivator 1 alpha	F: 5'-TGAAAGGGCCAAACAGAGAG-3'
		R: 5'-GTAAATCACACGGCGCTCTT-3'
NRF1	Nuclear respiratory factor 1	F: 5'-GGTGGGGGACAGATAGTCCT-3'
		R: 5'-ATGCTCACAGGGATCTGGAC-3'
PPARα	Peroxisome proliferator activated receptor alpha	F: 5'-CCGAGGGCTCTGTCATCA-3'
		R: 5'-GGGCAGCTGACTGAGGAA-3'
ERRα	Estrogen related receptor, alpha	F: 5'-CCTTCCCTGCTGGACCTC-3'
		R: 5'-CGACACCAGAGCGTTCACT-3'
ERRγ	Estrogen-related receptor gamma	F: 5'-AAGTGGGCATGCTGAAAGAA-3'
		R: 5'-CAGCATCTATTCTGCGCTTG-3'
Bcl-2	B cell leukemia/lymphoma 2	F: 5'-GTACCTGAACCGGCATCTG-3'
		R: 5'-GCTGAGCAGGGTCTTCAGAG-3'
Bax	BCL2 associated X, apoptosis regulator	F: 5'-GAACCATCATGGGCTGGA-3'
		R: 5'-GGTCCCGAAGTAGGAGAGGA-3'
Bak-1	BCL2-antagonist/killer 1	F: 5'-GGAATGCCTACGAACTCTTCA-3'
		R: 5'-CCAGCTGATGCCACTCTTAAA-3'
Bhmt	Betaine--homocysteine S-methyltransferase	F: 5'-GACAAGCTGGAAAACAGAGGA-3'
		R: 5'-CGTGCAATGTCACAAGCAG-3'
Otc	Ornithine carbamoyltransferase	F: 5'-TTCTCCACGGTCATTAGTGTTC-3'

		R: 5'-TCAGCAGGGATACCATGACA-3'
Acta1	Actin alpha 1, skeletal muscle	F: 5'-AATGAGCGTTTCCGTTGC-3'
		R: 5'-ATCCCCGCAGACTCCATAC-3'
mTOR	Mechanistic target of rapamycin kinase	F: 5'-AGGAGACCAGGGCCAAAG-3'
		R: 5'-AAGCGAGTAGACTCCTCCTGAC-3'
Pax7	Paired box 7	F: 5'-GGGGACAGAGGAAGATGCTA-3'
		R: 5'-GAGTGCTTAAGAAAGGTGCTTTG-3'
MyoD1	Myogenic differentiation 1	F: 5'-AGCACTACAGTGGCGACTCA-3'
		R: 5'-GGCCGCTGTAATCCATCAT-3'
Myog	Myogenin	F: 5'-CCTTGCTCAGCTCCCTCA-3'
		R: 5'-TGGGAGTTGCATTCACTGG-3'
Il6	Interleukin 6	F: 5'-GCTACCAAACTGGATATAATCAGGA-3'
		R: 5'-CCAGGTAGCTATGGTACTCCAGAA-3'
Tnfα	tumor necrosis factor alpha	F: 5'-TCTTCTCATTCCTGCTTGTGG-3'
		R: 5'-GAGGCCATTTGGGAACTTCT-3'
Mstn	Myostatin	F: 5'-TGGCCATGATCTTGCTGTAA-3'
		R: 5'-CCTTGACTTCTAAAAAGGGATTCA-3'
MuRF1	Muscle RING-finger protein-1	F: 5'-GGAAGTGTGCCAACGACAT-3'
		R: 5'-TCCAGACATGGACACTGAGC-3'
MAFbx	Muscle atrophy F-box	F: 5'-TCAAAGGCCTCACGATCAC-3'
		R: 5'-TCAGCCTCTGCATGATGTTC-3'
Col1a1	Collagen, type I, alpha 1	F: 5'-CATGTTCAGCTTTGTGGACCT-3'
		R: 5'-GCAGCTGACTTCAGGGATGT-3'
Col1a2	Collagen, type I, alpha 2	F: 5'-CAAGCATGTCTGGTTAGGAGAG-3'
		R: 5'-AGGACACCCCTTCTACGTTGT-3'
Col3a1	Collagen, type III, alpha 1	F: 5'-TTCCAGGACAACCAGGTCTC-3'
		R: 5'-AGTCGAATTGGGGAGAATAATTT-3'
Cdkn1a	Cyclin-dependent kinase inhibitor 1A	F: 5'-TCCACAGCGATATCCAGACA-3'
		R: 5'-GGACATCACCAGGATTGGAC-3'
Serpine1	Serine peptidase inhibitor, clade E, member 1	F: 5'-AGGATCGAGGTAAACGAGAGC-3'
		R: 5'-GCGGGCTGAGATGACAAA-3'
CD44	CD44 antigen	F: 5'-TGAGGAGACCTCAGATTCCAG-3'
		R: 5'-ACTATTGACCGCGATGCAG-3'
Myc	Myelocytomatosis oncogene	F: 5'-TTTGTCTATTTGGGGACAGTGTT-3'
		R: 5'-CATCGTCGTGGCTGTCTG-3'
Axin2	Axin 2	F: 5'-TGCAAAAGCCACCCAAAG-3'
		R: 5'-TTTTGGCAAGGTACCACCTC-3'

Hnf1a	HNF1 homeobox A	F: 5’-CCTCTCTCCCAGTAAGGTCCA-3’
		R: 5’-GCCGCAGACACTGTGACTAA-3’
Ccnd1	Cyclin D1	F: 5’-TTTCTTTCCAGAGTCATCAAGTGT-3’
		R: 5’-TGACTCCAGAAGGGCTTCAA-3’
Jun	Jun proto-oncogene	F: 5’-CTCCAAGTGCCGGAAAAG-3’
		R: 5’-GCGCTTTCAAGGTTTTCACT-3’
Hes5	Hes family bHLH transcription factor 5	F: 5’-CCCAAGGAGAAAAACCGACT-3’
		R: 5’-TGCTCTATGCTGCTGTTGATG-3’

Primers for qPCR with SYBR® Green system

Official Symbol	Name	Sequence
Hprt1	Hypoxanthine phosphoribosyltransferase 1	F: 5’-TGAAAGACTTGCTCGAGATGTCA-3’
		R: 5’-CACACAGAGGGCCACAATGT-3’
Fgf21	Fibroblast growth factor 21	F: 5’-CTGGGGGTCTACCAAGCATA-3’
		R: 5’-CACCCAGGATTTGAATGACC-3’
Mthfd2	methylenetetrahydrofolate dehydrogenase (NAD+ dependent), methenyltetrahydrofolate cyclohydrolase	F: 5’-AGGTCCCAAGCCTTTGAGTT-3’
		R: 5’-GTAAGGGAGTGCCGTTGAAA-3’
Gdf15	Growth differentiation factor 15	F: 5’-AGTGTCCCCACCTGTATCG-3’
		R: 5’-TGTCCTGTGCATAAGAACCA-3’

Supplementary Table 5

Supplementary Table 5. Antibody list. This table lists all of the antibodies used in this manuscript, including using on Western blots, Immunostaining (IHC/IF) and FASC sorting.

Antibodies for Western blots

Target protein	Manufacturer	Cat.no	Concentration
RRM2B	Abnova	PAB12860	1:1000
p53R2	GeneTex	GTX109620	1:1000
p53R2	abcam	ab8105	1:1000
HSPA1A	GeneTex	GTX111088	1:5000
RRM2	GeneTex	GTX103193	1:1000

Antibodies for IHC staining

Target protein	Manufacturer	Cat.no	Concentration
p53R2	GeneTex	GTX109620	1:500
Perilipin	Cell Signaling	3470	1:100

Antibodies for IF staining

Target protein	Manufacturer	Cat.no	Concentration
PAX7	abcam	ab34360	1:500
Pax-7 (PAX7)	Santa Cruz	sc-81648	1:500
Laminin	abcam	ab11575	1:500
Myh7	DSHB	BA-F8	1:200
Myosin heavy chain type IIA	DSHB	SC-71	1:200
Myosin heavy chain type IIB	DSHB	BF-F3	1:200
Myh1	DSHB	6H1	1:200
Heavy chain Myosin/MYH3	abcam	ab124205	1:500

Secondary antibodies for detection

Antibody	Manufacturer	Cat.no	Concentration
Goat anti-Rabbit IgG (H+L) Secondary Antibody, Alexa Fluor®488 conjugate	Thermo Fisher Scientific	A-11008	1:200
Goat anti-Mouse IgG (H+L) Secondary Antibody, Alexa Fluor®488 conjugate	Thermo Fisher Scientific	A-11001	1:200
Goat anti-Mouse IgG (H+L)	Thermo Fisher Scientific	A-11004	1:200

Secondary Antibody, Alexa Fluor®568 conjugate			
Goat anti-Rabbit IgG (H+L) Secondary Antibody, Alexa Fluor®568 conjugate	Thermo Fisher Scientific	A-11011	1:200
Alexa Flour® 350 IgG_{2b}	Thermo Fisher Scientific	A21140	1:200
Alexa Flour® 488 IgG_{1a}	Thermo Fisher Scientific	A21121	1:200
Alexa Flour® 555 IgG_{1a}	Thermo Fisher Scientific	A21426	1:200

Antibodies for FACS sorting

**Antibody**	**Manufacturer**	**Cat.no**
PE Rat Anti-Mouse CD106	BD Pharmingen™	561613
PE Rat IgG2a, **κ** Isotype Control	BD Pharmingen™	553930
BV786 Rat Anti-Mouse CD106-Clone 429	BD Pharmingen™	740865
BV786 Rat IgG2a, **κ** Isotype Control	BD Pharmingen™	563335
V500 Rat Anti-Mouse Ly-6A/E	BD Pharmingen™	561229
APC Rat Anti-Mouse CD31	BD Pharmingen™	561814
APC Rat Anti-Mouse CD45	BD Pharmingen™	561018
7-AAD	BD Pharmingen™	559925
