# Supplementary material for: Ribonucleotide reductase M2B in the myofibers modulates stem cell fate in skeletal muscle
Source: NPJ Regen Med. 2022 Jul 29;7:37. doi: 10.1038/s41536-022-00231-w (PMC9338274; doi:10.1038/s41536-022-00231-w)
Supplement: Supplementary file 2 — REPORTING SUMMARY [file 41536_2022_231_MOESM2_ESM.pdf]

nature portfolio

Corresponding author(s): Professor Yi-Fan Chen

Last updated by author(s): Jan 22, 2022

Reporting Summary

Nature Portfolio wishes to improve the reproducibility of the work that we publish. This form provides structure for consistency and transparency in reporting. For further information on Nature Portfolio policies, see our Editorial Policies and the Editorial Policy Checklist.

Statistics

For all statistical analyses, confirm that the following items are present in the figure legend, table legend, main text, or Methods section.

n/a	Confirmed	
☐	☒	The exact sample size (*n*) for each experimental group/condition, given as a discrete number and unit of measurement
☐	☒	A statement on whether measurements were taken from distinct samples or whether the same sample was measured repeatedly
☐	☒	The statistical test(s) used AND whether they are one- or two-sided<br>*Only common tests should be described solely by name; describe more complex techniques in the Methods section.*
☐	☒	A description of all covariates tested
☐	☒	A description of any assumptions or corrections, such as tests of normality and adjustment for multiple comparisons
☐	☒	A full description of the statistical parameters including central tendency (e.g. means) or other basic estimates (e.g. regression coefficient) AND variation (e.g. standard deviation) or associated estimates of uncertainty (e.g. confidence intervals)
☐	☒	For null hypothesis testing, the test statistic (e.g. *F*, *t*, *r*) with confidence intervals, effect sizes, degrees of freedom and *P* value noted<br>*Give P values as exact values whenever suitable.*
☐	☒	For Bayesian analysis, information on the choice of priors and Markov chain Monte Carlo settings
☐	☒	For hierarchical and complex designs, identification of the appropriate level for tests and full reporting of outcomes
☐	☒	Estimates of effect sizes (e.g. Cohen's *d*, Pearson's *r*), indicating how they were calculated

Our web collection on statistics for biologists contains articles on many of the points above.

Software and code

Policy information about availability of computer code

Data collection	Quantification of muscle fiber size was calculated by ImageJ software. Quantification of muscle fiber type distribution was analyzed by MetaXpress software. Flow cytometry and sorting data were analyzed with FACS Diva and FlowJo software.
Data analysis	Graphs and statistics were prepared using GraphPad Prism version 6 software or Microsoft Excel. Quantification of muscle fiber size was calculated by ImageJ software. Quantification of muscle fiber type distribution was analyzed by MetaXpress software. Quantification of Ki-67 IHC staining was calculated by MetaXpress software. Flow cytometry and sorting data were analyzed with FACS Diva and FlowJo software. The raw sequencing reads were processed using Cutadapt [v 1.16] to trim the adapter sequences and low-quality bases. The processed reads were then mapped to the mouse assembly GRCm38 using the STAR 2-pass mode [v 2.6.1a]. Gene expression quantification was performed using RSEM [v 1.2.31] with GENCODE annotations (release M18). Differential gene expression analysis was performed using the quasi-likelihood (QL) F-test method by edgeR.<br>Hierarchical clustering of selected groups of genes was performed in R, and the results were visualized as heatmaps using ComplexHeatmap. Pathway enrichment analysis was performed using IPA (Qiagen, Inc., Valencia, CA, USA) and enrichR (https://CRAN.R-project.org/package=enrichR), which is an R Interface to the Enrichr resource.

For manuscripts utilizing custom algorithms or software that are central to the research but not yet described in published literature, software must be made available to editors and reviewers. We strongly encourage code deposition in a community repository (e.g. GitHub). See the Nature Portfolio guidelines for submitting code & software for further information.

Data

Policy information about availability of data

All manuscripts must include a data availability statement. This statement should provide the following information, where applicable:

- Accession codes, unique identifiers, or web links for publicly available datasets
- A description of any restrictions on data availability
- For clinical datasets or third party data, please ensure that the statement adheres to our policy

The RNA-seq data have been deposited in the ArrayExpress database at EMBL-EBI (http://www.ebi.ac.uk/arrayexpress) under accession number E-MTAB-11318.

Field-specific reporting

Please select the one below that is the best fit for your research. If you are not sure, read the appropriate sections before making your selection.

☒ Life sciences ☐ Behavioural & social sciences ☐ Ecological, evolutionary & environmental sciences

For a reference copy of the document with all sections, see nature.com/documents/nr-reporting-summary-flat.pdf

Life sciences study design

All studies must disclose on these points even when the disclosure is negative.

Sample size	No statistical methods were used to predetermine sample size.
Data exclusions	No data was excluded from the analyses.
Replication	All attempts at replication were successful.
Randomization	The experiments were not randomized.
Blinding	The investigators were not blinded to allocation during experiments and outcome assessment.

Reporting for specific materials, systems and methods

We require information from authors about some types of materials, experimental systems and methods used in many studies. Here, indicate whether each material, system or method listed is relevant to your study. If you are not sure if a list item applies to your research, read the appropriate section before selecting a response.

Materials & experimental systems

n/a	Involved in the study	
☐	☒	Antibodies
☒	☐	Eukaryotic cell lines
☒	☐	Palaeontology and archaeology
☐	☒	Animals and other organisms
☒	☐	Human research participants
☒	☐	Clinical data
☒	☐	Dual use research of concern

Methods

n/a	Involved in the study	
☒	☐	ChIP-seq
☐	☒	Flow cytometry
☒	☐	MRI-based neuroimaging

Antibodies

Antibodies used

Antibodies for Western blots: RRM2B (Abnova, PAB12860), p53R2 (GeneTex, GTX109620), p53R2 (abcam, ab8105), HSPA1A (GeneTex, GTX111088)
Antibodies for IHC staining: p53R2 (GeneTex, GTX109620), Perilipin (Cell Signaling, 3470)
Antibodies for IF staining: PAX7(abcam, ab34360), Pax-7 (Santa Cruz, sc-81648), Myh7 (DSHB, BA-F8), Myosin heavy chain type IIA (DSHB, SC-71), Myosin heavy chain type IIB (DSHB, BF-F3), Myh1 (DSHB, 6H1), Goat anti-Rabbit IgG (H+L) Secondary Antibody, Alexa Fluor®488 conjugate (Thermo Fisher Scientific, A-11008), Goat anti-Mouse IgG (H+L) Secondary Antibody, Alexa Fluor®488 conjugate (Thermo Fisher Scientific, A-11001), Goat anti-Mouse IgG (H+L) Secondary Antibody, Alexa Fluor®568 conjugate (Thermo Fisher Scientific, A-11004), Goat anti-Rabbit IgG (H+L) Secondary Antibody, Alexa Fluor®568 conjugate (Thermo Fisher Scientific, A-11011), Alexa Flour® 350 IgG2b (Thermo Fisher Scientific,A21140), Alexa Flour® 488 IgG1a (Thermo Fisher Scientific, A21121), Alexa Flour® 555 IgG1a (Thermo Fisher Scientific, A21426)
Antibodies for FACS sorting: PE Rat Anti-Mouse CD106 (BD Pharmingen™, 561613), PE Rat IgG2a, ? Isotype Control (BD Pharmingen™, 553930), BV786 Rat Anti-Mouse CD106-Clone 429, BD Pharmingen™, 740865), BV786 Rat IgG2a, ? Isotype Control (BD

	Pharmingen™, 563335), V500 Rat Anti-Mouse Ly-6A/E (BD Pharmingen™, 561229), APC Rat Anti-Mouse CD31 (BD Pharmingen™, 561814), APC Rat Anti-Mouse CD45 (BD Pharmingen™, 561018), 7-AAD (BD Pharmingen™, 559925)
Validation	All the antibodies which were used in this manuscript are commercial products purchased from the manufacturer. The validation statements and further citations can be found on the manufacturers' websites according to the antibodies' cat. numbers.

Animals and other organisms

Policy information about studies involving animals; ARRIVE guidelines recommended for reporting animal research

Laboratory animals	B6.Cg-Tg(ACTA1-cre)79Jme/J (stock number: 006149), B6.CgPax7tm1(cre/ERT2)Gaka/J mice (stock number: 017763), and Gt(ROSA)26Sortm4(ACTBtdTomato,EGFP)Luo/J (ROSAmT/mG, stock number: 003474) mice were purchased from Jackson lab to establish genetically modified mouse models. Male mice from 3 to 24 months old were used for analysis.
Wild animals	The study did not involve wild animals.
Field-collected samples	The study did not involve sample collected from the field.
Ethics oversight	All animal protocols were approved by the Institutional Animal Care and Use Committee of Taipei Medical University and the National Defense Medical Center.

Note that full information on the approval of the study protocol must also be provided in the manuscript.

Flow Cytometry

Plots

Confirm that:

☒ The axis labels state the marker and fluorochrome used (e.g. CD4-FITC).

☒ The axis scales are clearly visible. Include numbers along axes only for bottom left plot of group (a 'group' is an analysis of identical markers).

☒ All plots are contour plots with outliers or pseudocolor plots.

☒ A numerical value for number of cells or percentage (with statistics) is provided.

Methodology

Sample preparation	Briefly, hindlimb muscles were collected, and digested using Collagenase II (Worthington) and Dispase II (Thermo Fisher). MuSCs were then dissociated from the myofibers with a 20G needle, and the resulting cellular suspension was filtered using 40-µm cell strainers.
Instrument	FACSVerse was used for flow cytometry, and FACS Aria Fusion was used for cell sorting.
Software	The authors used the FACS Diva and FlowJo software to collect and analyze the flow cytometry data.
Cell population abundance	The activated muscle stem cells are about 5% of all cell populations.
Gating strategy	We followed the protocol published in Nature Protocol in 2015. (Nat Protoc . 2015 Oct;10(10):1612-24.)First we used the FSC/SSC gating to identify the single cell population. According to the cell surface's markers, we gated those cells showing CD31-/CD45-/Sca1-/Vcam+ to isolate the group of muscle stem cells.

☒ Tick this box to confirm that a figure exemplifying the gating strategy is provided in the Supplementary Information.
